# Quantitative Phosphoproteomic Analysis Reveals Dendritic Cell- Specific STAT Signaling After α2-3–Linked Sialic Acid Ligand Binding

**DOI:** 10.3389/fimmu.2021.673454

**Published:** 2021-04-22

**Authors:** Rui-Jún Eveline Li, Aram de Haas, Ernesto Rodríguez, Hakan Kalay, Anouk Zaal, Connie R. Jimenez, Sander R. Piersma, Thang V. Pham, Alex A. Henneman, Richard R. de Goeij-de Haas, Sandra J. van Vliet, Yvette van Kooyk

**Affiliations:** ^1^ Department of Molecular Cell Biology and Immunology, Cancer Center Amsterdam, Amsterdam Infection and Immunity Institute, Amsterdam UMC, Vrije Universiteit Amsterdam, Amsterdam, Netherlands; ^2^ Department of Medical Oncology, Cancer Center Amsterdam, Amsterdam UMC, Vrije Universiteit Amsterdam, Amsterdam, Netherlands

**Keywords:** dendritic cell, sialic acid, α2-3sia, STAT3, STAT5, Phosphoproteomics, Siglec, tolerance

## Abstract

Dendritic cells (DCs) are key initiators of the adaptive immunity, and upon recognition of pathogens are able to skew T cell differentiation to elicit appropriate responses. DCs possess this extraordinary capacity to discern external signals using receptors that recognize pathogen-associated molecular patterns. These can be glycan-binding receptors that recognize carbohydrate structures on pathogens or pathogen-associated patterns that additionally bind receptors, such as Toll-like receptors (TLRs). This study explores the early signaling events in DCs upon binding of α2-3 sialic acid (α2-3sia) that are recognized by Immune inhibitory Sialic acid binding immunoglobulin type lectins. α2-3sias are commonly found on bacteria, e.g. Group B *Streptococcus*, but can also be expressed by tumor cells. We investigated whether α2-3sia conjugated to a dendrimeric core alters DC signaling properties. Through phosphoproteomic analysis, we found differential signaling profiles in DCs after α2-3sia binding alone or in combination with LPS/TLR4 co-stimulation. α2-3sia was able to modulate the TLR4 signaling cascade, resulting in 109 altered phosphoproteins. These phosphoproteins were annotated to seven biological processes, including the regulation of the IL-12 cytokine pathway. Secretion of IL-10, the inhibitory regulator of IL-12 production, by DCs was found upregulated after overnight stimulation with the α2-3sia dendrimer. Analysis of kinase activity revealed altered signatures in the JAK-STAT signaling pathway. PhosphoSTAT3 (Ser727) and phosphoSTAT5A (Ser780), involved in the regulation of the IL-12 pathway, were both downregulated. Flow cytometric quantification indeed revealed de- phosphorylation over time upon stimulation with α2-3sia, but no α2-6sia. Inhibition of both STAT3 and -5A in moDCs resulted in a similar cytokine secretion profile as α-3sia triggered DCs. Conclusively, this study revealed a specific alteration of the JAK-STAT pathway in DCs upon simultaneous α2-3sia and LPS stimulation, altering the IL10:IL-12 cytokine secretion profile associated with reduction of inflammation. Targeted control of the STAT phosphorylation status is therefore an interesting lead for the abrogation of immune escape that bacteria or tumors impose on the host.

## Introduction

Dendritic cells (DCs) are antigen presenting cells that continuously sense the intrinsic host environment. DCs possess the extraordinary capacity to recognize internal and external danger signals and respond appropriately using pattern recognition receptors (PPRs) (1). Upon encounter of pathogen-associated molecular patterns (PAMPs) or danger-associated molecular patterns (DAMPs), DCs can leave the periphery and migrate to the lymphoid tissues to activate an appropriate adaptive immune response (2). In contrast, recognition of self-associated molecular patterns (SAMPs), such as self-antigens, leads to the induction of a tolerogenic response (3).

Glycans form cellular immune recognition elements and are considered key modulators of the immunological outcome (4, 5). Glycosylation is a common post-translational modification in eukaryotes, and glycan patterns can be recognized as PAMPs or SAMPs by DC PRRs, such as C-type lectin receptors (CLRs) and Sialic acid binding immunoglobulin type lectins (Siglecs) (6). SAMP-associated glycan patterns predominantly refer to sialic acids (3). This negatively charged monosaccharide decorates the terminal positions of larger polysaccharide molecules on cell surfaces. Positioned at the outer rim of the glycocalyx in an α2-3-, α2-6-, or α2-8-linkage, sialic acids portray a dominant role in cell-cell interactions and maintenance of intrinsic homeostasis (7). Due to its presence on all cells, the inherent sialic acid signature is an effective marker to promote tolerance upon encounter of self-antigens (3). Synthetic antigens modified with a sialic acid can alter the immunogenicity of the antigen by imposing a regulatory program on DCs. The DC can subsequently skew the differentiation of naïve T cells to regulatory T cells *via* e.g. an altered cytokine secretion profile, and reduce inflammatory T effector cell responses (8). The impact of sialic acids on altering T cell differentiation is therefore highly appealing as a target in DC-based immunotherapeutic strategies (9, 10).

Furthermore, sialic acids are increasingly acknowledged for their role in the immune regulation of cancer. During cancer progression, tumor cells often highly increase their sialic acid expression to create an immunosuppressive tumor microenvironment (11). Tumor hypersialylation furthermore alters myeloid cells and hamper immunotherapy efficacy, as the T cell and NK cell responses are dampened by the tolerogenic immune signals emanating from the tumor cell surfaces (11). Novel approaches to combat quenching of immune cell activity using targeted delivery of sialyltransferase inhibitors are currently being explored to improve immunotherapeutic strategies (12, 13).

Sialic acids are also taken advantage of by pathogens to benefit their own survival. Bacteria obtain sialic acids by *de novo* synthesis or from an environmental source (14). By doing so pathogens can hide and escape from immune surveillance. Group B *Streptococcus* (GBS) uses sialic acids to mimic the host cell surface. The capsular polysaccharides of all serotypes are decorated with terminal α2-3-linked sialic acids, causing suppression of the host immune response, promoting bacterial survival (15, 16). This exploitation of sialic acid by GBS eventually results in the devastatingly high incidence of sepsis and meningitis in infants (16).

To gain insight into the self and foreign discrimination by DCs, and the *in vivo* induction of an immune suppressive T cell response (8), we explored human DC immune signaling upon sialic acid binding. We conjugated α2-3-linked sialic acids to a dendrimeric core for multivalent ligand presentation. By proteomic and phosphoproteomic analysis we studied the induced signaling pathways. With concomitant TLR4 stimulation to trigger DC maturation and cytokine secretion, we mimicked bacterial pathogen recognition by DCs. We report specific signaling profiles upon stimulation with α2-3 sialic acid in presence of LPS, affecting kinases within the MAPK/ERK and JAK-STAT pathway and subsequent anti-inflammatory cytokine responses. These results demonstrate the dynamic signaling networks and specific pathways underlying DC immune suppressive signaling upon recognition of sialic acid linkages.

Targeted control of STAT phosphorylation provides an interesting lead for the revocation of tolerance in bacterial and tumoral immune surveillance escape. Continued investigations on the DC signaling cascade from the JAK-STAT pathway to the control of the IL-12 transcripts and the ensuing suppression of effector T cells could be an appropriate continuation of this study. Moreover, this study also provided insight in the alterations in the MAPK signaling pathways and other kinome signatures, which were not pursued here. Further efforts to analyze these pathways and profiles will yield information on the role of DCs in the polarization of naïve T cells towards effector or regulatory T cells. The upstream signaling from the α2-3sia binding Siglec towards the immunosuppressive DC phenotype has important practical implications for the use of the sialic acid-Siglec axis as a therapeutic strategy in immunotherapy. The homology of the Siglec receptors and their affinity towards multiple sialic acids complicates their therapeutic application. Moreover, it must also be taken into account that the use of Siglec receptor-specific antibodies as blocking agent may also trigger Siglec-dependent signaling pathways. Nonetheless, these challenges are manageable with the rapid developments currently in the field of Siglec research.

## Materials and Methods

### Synthesis of the Glycodendrimers

Three glycodendrimers were synthesized for this study, the control, α2-3 sialic acids and α2-6 sialic acids. To generate 2.0 PAMAM dendrimers with a cystamine core (Sigma-Aldrich) the glycans 3’-Sialyl-N-acetyllactosamine (Dextra Laboratories; α2-3sia dendrimer) and 6’-Sialyl-N-acetyllactosamine (Dextra Laboratories; α2-6sia dendrimer), and D-(+)-galactose (Sigma-Aldrich; control dendrimer), were conjugated *via* reductive animation using the free reducing ends. Approximately 32 equivalents of the glycan were added per dendrimer in dimethylsulphoxide (DMSO, Sigma-Aldrich) and acetic acid (8:2 ratio, Sigma-Aldrich). To the cocktail 160 equivalents of 2-Methylpyridine borane complex (Sigma-Aldrich) was added up to a desired total volume of 200 µL, and incubated at 65°C for 2 hours with repeated vortexing. The reaction products were purified over disposable PD10 desalting columns (GE Healthcare) using 50 mM NH_4_HCO_3_ pH 4.4, and submitted to multiple cycles of lyophilization and redisolving in H_2_O. The products were validated using LC-MS and plant lectin binding.

### Primary Cell Isolation and Culture

Monocytes were obtained from buffy coats obtained from healthy donors (Sanquin Amsterdam, reference: S03.0023-XT) using Ficoll (Stemcell Technologies) and Percoll (Sigma-Aldrich) gradient centrifugation. The monocytes were cultured for four days in RPMI 1640 (Invitrogen), supplemented with 10% FCS (Biowittaker), 1.000 U/mL penicillin (Lonza), 1 U/mL streptomycin (Lonza), 262.5 U/mL IL-4 (Biosource) and 112.5 U/mL GM-CSF (Biosource) to obtain immature monocyte-derived DCs (moDCs). Expression of CD1a and CD14 (both BioLegend) was monitored *via* flow cytometric analysis as markers of moDC differentiation, and CD83 and CD86 (both Becton Dickinson) as markers of maturation.

### Cytokine Analysis

1 µM dendrimer was added to approximately 50·10^3^ day 4 moDCs, with or without 10 ng/mL LPS derived from E. coli 0111:B4 (Sigma-Aldrich). For the inhibition studies, 0.25 µM of the STAT5 inhibitor CAS 285986-31-4 (Calbiochem) or the STAT3 inhibitor JSI-124 (Sigma Aldrich) were used. After overnight stimulation, the supernatants were harvested and the cytokines IL-10 and IL-12p70 (both Biosource) were measured by sandwich ELISA according to the manufacturer’s protocol. Briefly, NUNC MaxiSorp plates were coated with the capture antibody in 0.05 M NaHCO_3_ buffer overnight at 4°C. The plates were washed and blocked using PBS + 1% BSA (EMD Millipore). The supernatants were incubated on the coated plates for 2 hours at room temperature, washed, and binding of the cytokine was detected with a peroxidease-conjugated detection antibody. Binding was visualized with 3,3’,5,5’-tetramethylbenzidine (Sigma-Aldrich) and quantified on the iMarkTM Microplate Absorbance Reader (Bio-RAD) at 450 nm.

### Primary Cell Stimulation, Lysis and Protein Extraction

Approximately 2.5·10^7^ day 4 moDCs were stimulated at 37°C with 1 µM of the dendrimer with or without LPS (Sigma-Aldrich). After 30 minutes, the cells were immediately cooled to 4°C by placement on ice and washed using pre-cooled 4°C PBS. Lysis buffer (20 mM HEPES pH 8.0, 9 M CH_4_N_2_O, 1 mM Na_3_VO_4_, 2.5 mM Na_4_P_2_O_7_, and 1 mM Na_2_C_3_H_7_PO_6_) was freshly prepared and added to the cells. After vortexing the cells were snap frozen in liquid nitrogen. The protein concentration was measured by BCA assay (Thermo Fisher Scientific) according to manufacturer’s protocol. 45 mM DDT was added to 10 mg protein, incubated for 30 minutes at 55°C, followed by reduction of the lysate. Subsequent addition of 110 mM iodoacetamide solution alkylated the protein lysate. The urea concentration was then diluted to 2 M with 20 mM HEPES buffer pH 8.0 for digestion with sequencing grade modified trypsin (enzyme:protein 1:100 w/w). The tryptic lysate digests were acidified with 1% TFA and checked for the pH (<3). The tryptic peptides were then captured through solid-phase extraction with the OASIS HLB-based cartridges (Waters Corporation). After washing with 0.1% TFA, the peptides were eluted with 0.1% TFA and 80% acetonitrile.

### TiOx Phosphopeptide Enrichment

Titanium dioxide (TiOx) chromatography was applied to capture the phosphopeptides. 500 µg desalted tryptic digests were diluted 1:1 with lactic acid solution (0.3 g/mL lactic acid, 0.07% TFA/53% acetonitrile). 200 µL pipette tips were fitted with a 16G-needle punch of a C8 disk EMPORE, on which 2.5 mg TiO_2_ was added. The TiOx bed was preconditioned with 0.1% TFA and 80% acetonitrile before equilibration with 0.3 g/mL lactic acid in 0.07% TFA/54% acetonitrile, allowing capture of phosphorylated serine and threonine peptides of the tryptic digest. After sequential washing of the bedding with lactic acid, and 0.1% TFA + 80% acetonitrile, the phosphopeptides were eluted with 0.5% and 5% (v/v) piperidine in 20% (v/v) phosphoric acid to quench the basic solution. Pipette tips (200 µL) were again fitted with a 16G-needle punch of an EMPORE disk of poly(StyreneDivinylBenzene) material, preconditioned with 0.1% TFA and 80% acetonitrile, and equilibrated with 0.1% TFA. After loading the enriched phosphopeptide mixture, the bedding was washed with 0.1% TFA. Through centrifugal filtration, the phosphopeptides were desalted in 0.1% TFA and 80% acetonitrile and lyophilized.

### NanoLC-MS/MS Acquisition and Data Processing

The peptides were redissolved in loading solvent (0.5% TFA/4% acetonitrile) prior to separation on an Ultimate 3000 nanoLC (Dionex LC-Packings) equipped with a 20 cm x 75 µmID fused silica column, custom packed with 3 µm 120 Å ReproSil Pur C18 aqua (Dr Maisch GMBH) on-line coupled to a MS/MS platform (QExactive, ThermoFisher). The MS/MS spectra were matched to the Uniprot human reference proteome FASTA file (release February 2013, 70136 entries) in MaxQuant v1.4.1.2. The measured phosphopeptides intensities were normalized to the median intensity of all identified peptides (‘normalized intensity’ from the MaxQuant Evidence table) and quantified by their extracted ion chromatograms (‘Intensity’ in MaxQuant). The fold change was calculated in R, as well as the p values from the replicates using a limma test (17), which were considered significantly altered at p < 0.05. Phosphopeptide quantification by the OncoProteomics Laboratory, VUmc has been previously described thoroughly (18, 19). The significant peptides were functionally correlated using the online STRING tool v11.0 (https://string-db.org/), and mapped in Cytoscape v3.5.1 (https://cytoscape.org/) (20, 21). Gene Ontology (GO) term enrichment analysis was performed with the Cytoscape plugin ClueGO v2.5.5 (http://apps.cytoscape.org/apps/cluego) (21). The significantly altered phosphoproteins were integrated and visualized in pathways using Pathview under default settings (https://pathview.uncc.edu/) (22). Phosphoproteomic alterations were analyzed and visualized using Integrative Inferred Kinase Activity (InKA) analysis v1.2.2 (https://inkascore.org/) and PTMsigDB analysis v2.0 (https://github.com/broadinstitute/ssGSEA2.0) (23, 24).

### Flowcytometric Quantification of Phosphoproteins

Approximately 5·10^4^ day 4 moDCs were stimulated at 37°C with 1 µM the dendrimer with or without LPS (Sigma-Aldrich). After the indicated time points, cells were immediately cooled to 4°C by placement on ice and washed using pre-cooled 4°C PBS. The cells were gently fixed in PBS + 4% PFA, for 15 minutes at room temperature, followed by washing in PBS. Permeabilization of the cells was performed by adding 90% pre-cooled 4°C methanol for 30 minutes. After washing, STAT3 (1:1000, clone 124H6, Cell Signaling), STAT5A (1:500, clone 4H1, Cell Signalling), pSTAT3 (1:1000, clone E121-31, Abcam), or pSTAT5A (1:500, ab30649, Abcam) antibodies were added and incubated for 60 minutes at room temperature. After extensive washing, the secondary antibodies Alexa-488 goat anti-mouse IgG2a and Alexa-647 donkey anti-rabbit IgG (both 1:1000, from Invitrogen) were added for 30 minutes before flow cytometric analysis by CyAn™ ADP (Beckman Coulter), and analyzed using FlowJo v10.

### Statistics

The plotted data is represented as mean ± SD of at least three healthy donors or independent experiments. The statistical analyses were performed in GraphPad Prism v7.04. Independent samples were evaluated by the Students t-test, groups with a non-normal distribution were compared by the Kruskal-Wallis test, with the overall statistical significance set at P < 0.05.

## Results and Discussion

### Quantitative Analysis of the DC Phosphoproteome After α2-3 Sialic Acid Stimulation

**Figure 1:**
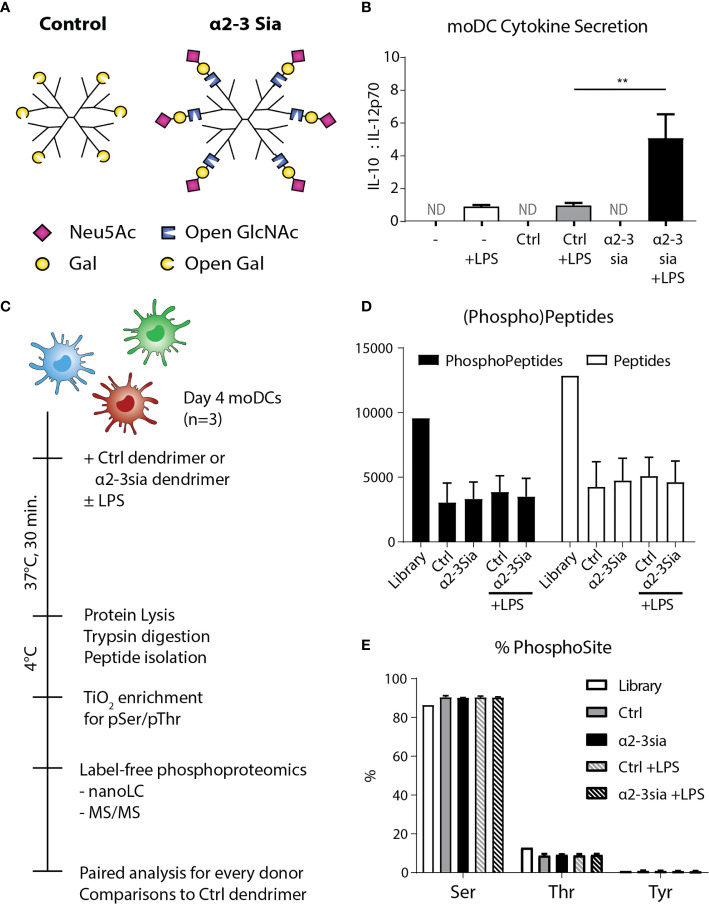
Experimental setup and quality control of the phosphoproteomics. **(A)** A schematic model of the two glycodendrimers synthesized, the control and α2-3 sialic acid dendrimer. Through reductive animation an excess of the glycans is introduced and conjugated to the dendrimer, by opening the saccharide ring at the reducing end. **p < 0.01 **(B)** After overnight stimulation of the moDCs with the dendrimers, IL-10 and IL-12 were quantified in the supernatant through an ELISA assay. Only in the conditions with LPS stimulation cytokines were measured. Stimulation with LPS plus the α2-3 sialic acid dendrimer resulted in a significant decrease of IL-10. One donor is depicted as a representative of 8 individuals, ND, Not Determined; range IL-10 319-6072 pg/mL; IL-12 48-1175 pg/mL. **(C)** Day 4 human moDCs from three independent donors were stimulated with the control or the α2-3 sialic acid dendrimers with or without LPS stimulation for 30 minutes at 37°C. The cells were immediately cooled to 4°C to maintain the phosphoprotein signature, and lysed. The lysate was digested with trypsin, and subsequently prepped and subjected for peptide isolation. Using TiOx chromatography, the lysate was enriched for phospho-serine and –threonine, before label-free phosphoproteomic analysis using LC-MS/MS. **(D)** 9,566 phosphopeptides and 12,851 proteins were quantified with a false discovery rate of < 1% (n=3). **(E)** Approximately 90% of the phosphopeptides identified were phosphorylated at the serine, 10% at the threonine, and <1% at the tyrosine (n=3).

To obtain a global overview of the α2-3 sialic acid-induced signaling in DCs, we performed LC-MS/MS-based phosphoproteomic analysis of human DCs stimulated with sialic acid-coated dendrimers. From peripheral blood of three human donors, we isolated the monocytes and differentiated these to monocyte-derived DCs (moDCs) using an IL-4/GM-CSF cocktail for four days of culture. We introduced α2-3-linked sialic acids (α2-3sia) to a dendrimer through reductive animation (25). The small second generation dendrimeric core was selected as carrier system, as the spherical platform allows compact packing of the glycans and multivalent presentation on the polymeric arms. We selected 3’-sialyl-N-acetyllactosamine for coupling, as the saccharide ring is opened at the carbon atom during conjugation (**Figure 1A**). By using this trisaccharide, the α2-3sia–galactose linkage is maintained, mimicking the sialic acid decorated bacterial capsule of GBS (26). We also conjugated a galactose in a similar fashion, which served as a C_6_H_12_O_5_ (open galactose)-dendrimer control. The dendrimers were validated using plant lectins (**Sl. Figure 1**) and incubated after overnight with moDCs to study alterations in cytokine secretion profiles. IL-12 secreted by DCs is a key inducer of the pro-inflammatory immune response, while IL-10 regulates IL-12 production and plays a significant role the induction of regulatory T cells (27). The antagonistic relation of the two cytokines is therefore an effective indicator of the DC immune status. We determined the optimal dendrimer concentration at 1 µM (data not shown) measured an increased IL-10:IL-12 secretion profile when the α2-3sia dendrimer was given to DCs in the presence of LPS, which was not observed with the control dendrimer (**Figure 1B**). No cytokines were measured in the medium controls, without the TLR4 stimulus.

To decipher the signaling events occurring upon α2-3 sialic acid recognition by dendritic cells, we added 1 µM of the glycodendrimer to approximately 2.5·10^7^ moDCs with or without LPS for 30 minutes at 37°C (**Figure 1C**). To maintain the phosphorylation signatures, the cells were immediately chilled, lysed in a buffer with protease and phosphatase inhibitors, and snap-frozen in liquid nitrogen until the solid-phase extraction of the peptides before titanium dioxide (TiOx) chromatography and nanoLC-MS/MS quantification. A total of 9,566 phosphopeptides and 12,851 proteins were quantified with a false discovery rate of < 1% (**Figure 1D**). The majority of the phosphopeptides found were phosphorylated at the serine, while only approximately 10% was phosphorylated at the threonine (**Figure 1E**). TiOx chromatography also captured the much less prevalent phosphorylated tyrosine residues, resulting in a total presence of <1%. The amount of (phospho)peptides and sites identified was not altered upon stimulation with either of the glycodendrimers.

### Alterations in the moDC Phosphoproteome After α2-3 Sialic Acid Binding

**Figure 2:**
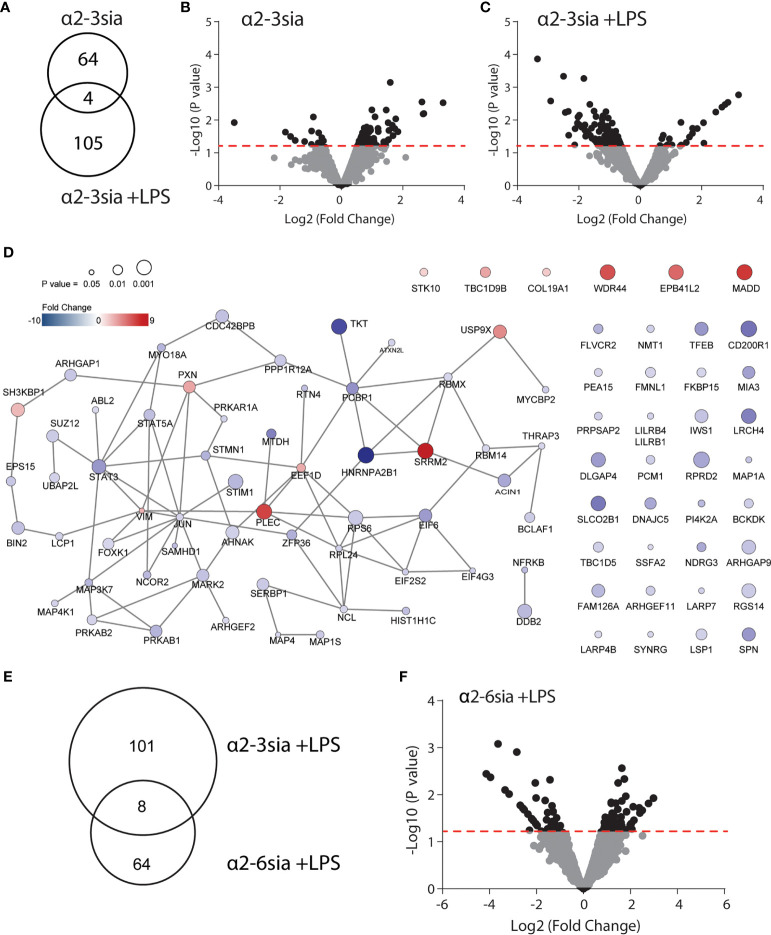
Alterations in the phosphoproteomic landscape after sialic acid stimulation. **(A)** A total of 68 phosphorylation sites were significantly altered by α2-3sia stimulation, while 109 were altered by α2-3sia and LPS. Only 4 phosphorylation sites were identical between the two conditions. **(B)** The Volcano plot demonstrates the directionality of the phosphorylation status on proteins after stimulation with the α2-3sia dendrimer. The data above de red dotted line represent all the significantly altered phosphoproteins in three donors. The –Log10 of the average p values, calculated with a limma test, is presented against the Log2 in fold change. **(C)** The Volcano plot demonstrates modified phosphoprotein expression after stimulation with the α2-3 sialic acid dendrimer. The data above de red dotted line represent all the significantly altered phosphoproteins in three donors. The –Log10 of the average p values, calculated with a limma test, is presented against the Log2 in fold change. **(D)** STRING analysis reveals a network of 88 phosphoprotein significantly affected by α2-3 sialic acid binding in the presence of LPS. **(E)** Phosphoproteomic analysis after α2-6sia stimulation in presence of LPS revealed 72 significantly altered phosphoproteins, of which 8 overlapped with α2-3sia stimulation with LPS. **(F)** The Volcano plot demonstrates the directionality of phosphorylation status on proteins after stimulation with the α2-6sia dendrimer and LPS. The data above de red dotted line represent all the significantly altered phosphoproteins in three donors. The –Log10 of the average p values, calculated with a limma test, is presented against the Log2 fold change.

To reveal the most significantly altered phosphoproteins, we used a pairwise comparison for each donor. Furthermore, the sialic acid stimulated conditions were compared to the ctrl-dendrimer stimulations (Ctrl *vs* α2-3sia; Ctrl+LPS *vs* α2-3sia+LPS). A total of 68 significantly altered phosphorylation sites were found upon α2-3 sialic acid triggering, while, compared to the control dendrimer, simultaneous α2-3sia and LPS stimulation resulted in 109 altered phosphosites (**Figures 2A–C** and **Sl. Tables 1, 2**). Only 4 altered phosphorylation sites were shared between the conditions (AHNAK Thr4100, Ser5749, Ser5393 and STK10 Ser448). The presence of LPS resulted in a different signaling profile, indicating modulation of the TLR4 signaling pathway by α2-3sia. The additional 41 altered phosphorylation sites found in the simultaneous α2-3sia and LPS-stimulated condition could therefore be involved in crosstalk between the α2-3sia dendrimers binding receptors and TLR4 signaling. The 86% decrease in phosphorylated proteins in the LPS stimulated condition is furthermore notable, while 84% of the phosphoproteins in the α2-3sia-dendrimer-only condition had a higher phosphorylation status (**Figure 2C** and **Sl. Tables 1, 2**). To determine whether the identified phosphoproteins are functionally cooperative after α2-3sia stimulation, sequential STRING and cytoscape analysis was employed on the significantly altered phosphoproteins (28). Due to the relatively low number of phosphoproteins, we excluded a cutoff in fold change and explored all 109 significantly altered phosphorylation sites. This resulted in a network of 58 significant phosphoproteins functionally interconnected after α2-3sia stimulation in presence of LPS (**Figure 2D** and **Sl. Table 2**). Only 7 of these phosphoproteins in the network were increased in phosphorylation. Remarkable were SRRM2 with the highest increase (9.19-fold) in phosphorylation, and HNRNPA2B1 with the strongest decrease in phosphorylation of 10.2-fold. SRMM2 is involved in pre-mRNA splicing, and HNRNPA2B1 is associated with packaging of pre-mRNAs into exosomes (28, 29). The two proteins are known to interact, suggesting a role for sialic acids in the processing of pre-mRNA (30). Furthermore, two STAT proteins had a lower phosphorylation status (4.08-fold decrease for STAT3, and 2.53-fold decrease for STAT5A). Both proteins have dual roles as signal transducers and transcription factors, and are key regulators of DC activity and DC skewing of specific T cell responses (31, 32). Notable is the phosphoprotein JUN with the most interactions with 9 other linked nodes. The 1.75-fold de-phosphosphorylated protein JUN is a transcription factor and interconnects with other DNA/RNA binding proteins, indicating activation of genetic reprogramming after α2-3sia stimulation. Analysis of altered phosphoproteins in the α2-3sia only conditions showed a small cluster of 13 interconnected nodes, and separate connections between 13 phosphoproteins (**Sl. Figure 2**). Notable is the connectivity between the SRRM2 protein with ACIN1. The mRNA splicing involved protein was upregulated in phosphorylation upon α2-3sia, while the presence of LPS downregulated phosphorylation on this protein. Furthermore, the phosphorylation of the transcriptional repressor BCLAF1 was remarkable. Stimulation with α2-3sia increased phosphorylation 2-folds at Ser285, while α2-3sia and LPS co-stimulation resulted in 2-fold downregulation at the same site (**Sl. Tables 1, 2**). In conclusion, upon α2-3sia stimulation phosphorylation was enhanced upon α2-3sia stimulation, while simultaneous α2-3sia and LPS stimulation resulted in less phosphorylation, indicating that recognition of a2-3 sialic acid by DC alters TLR 4 triggering and DC signaling. Differential signaling is therefore identified after α2-3sia stimulation in presence or absence of LPS, which leads, amongst others, to an altered cytokine secretion profile.

**Figure 3:**
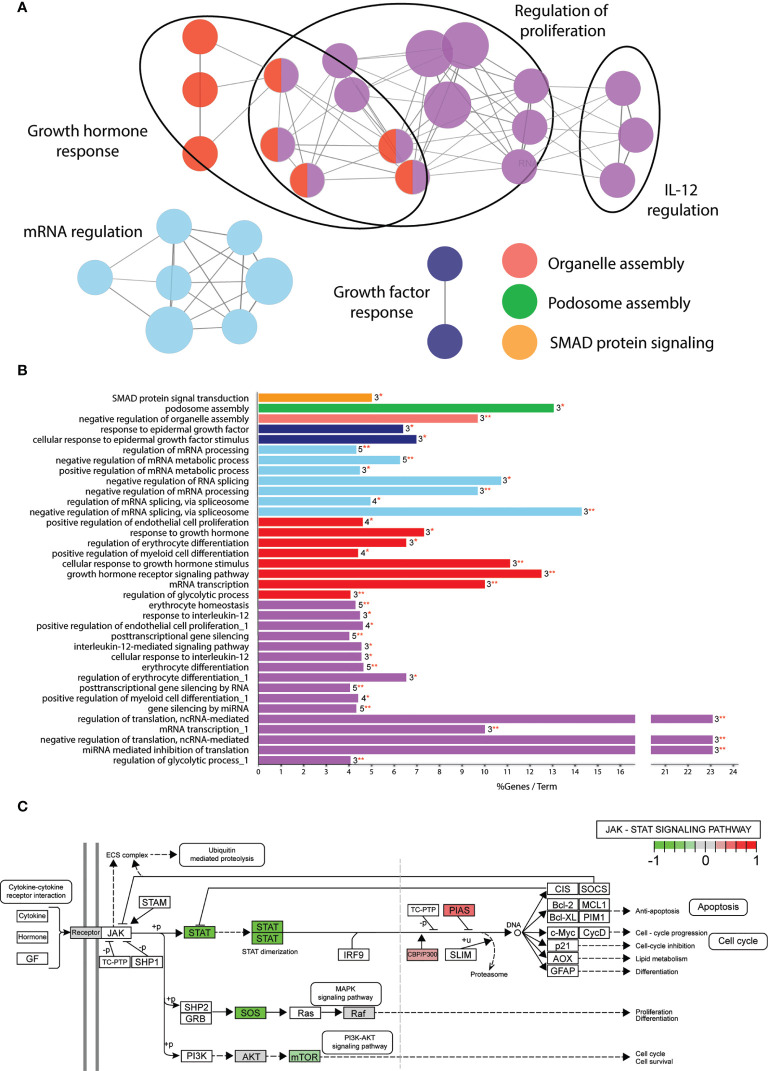
Significantly altered phosphoproteins are involved in multiple biological processes. **(A)** GO term enrichment analysis through ClueGO of the α2-3sia altered proteins in presence of LPS mapped to multiple networks. The node color groups multiple GO terms. The node size represents the number of genes annotated to each term, and de edges between the nodes indicate an overlap in proteins. The differentially affected proteins are clustered into multiple networks and processes. **(B)** GO analysis results are classified in functionally grouped network of terms/pathways and color coded to the GO groups. The bars represent the % genes per term, followed by the absolute number of proteins annotated to the term and with what significance (*p < 0.05, **p < 0.01). Terms with multiple occurrences in functional groups are marked with “_1” in the name. The differentially affected proteins are categorized into 7 groups total. **(C)** Visualization of the significantly altered proteins of α2-3sia stimulated moDCs in presence of LPS within the JAK-STAT signaling pathway.

To further investigate the specificity of the α2-3-linked sialic acid altered phosphorylation, we additionally synthesized dendrimers with α2-6-linked sialic acids (α2-6sia, **Sl. Figure 1**). Although both structures contain a terminal sialic acid, the linkage to the underlying galactose is structurally different. The α2-6sia binding to moDCs was studied in presence of LPS, as more biological processes were affected, similarly to the α2-3sia binding with LPS (**Figure 2E** and **Sl Figure 3A**). Only 8 of the 72 significantly altered phosphoproteins were shared between the two glycans in presence of LPS and phosphorylated at the at the same phosphorylation site (**Figures 2E, F** and **Table 1**). The directionality of the protein phosphorylation status additionally overlapped, except for RGS14 phosphorylation (-2.06 with α2-3sia; 2.27 with α2-6sia). Other studies have found a relationship between this G protein-coupled receptor and TLR4 signaling, where stimulation of DCs with LPS markedly decreased RGS14 phosphorylation, which negatively impacted DC IL-12 production (33, 34). To reveal whether the 8 overlapping phosphoproteins were correlated in function, we performed STRING analysis. However, no interactions were found (data not shown). Therefore, we concluded that the stimulation with α2-3sia leads to a very distinct signaling profile compared to α2-6sia in presence of LPS. The distinction between the two patterns might be explained by might be explained by the presence of multiple Siglec receptors and their individual binding preferences for specific sialic acid linkages (35). Siglec-7, and -9 are expressed by moDCs and bind α2-3sia, while α2-6sia is recognized by more Siglecs (36). Siglec-10 binds α2-6sia and is also able to recognize α2-3sia to a lesser extent (36). Nonetheless, the Siglec-10 receptor is expressed on moDCs only at very low levels compared to Siglec-7 and -9 (36). This would indicate binding of the α2-3sia dendrimer to both Siglec-7 and -9, while the α2-6sia dendrimer likely triggers other Siglec receptors. The observed signaling patterns are therefore the result of the collective Siglec receptors expressed by moDCs that recognize the particular sialic acid. Further elucidation of the Siglec-specific pathways upon α2-3sia and LPS co-stimulation would therefore be an intriguing area for future investigations. Analysis of altered biological processes after α2-3 sialic acid and LPS encounter.

**Table 1:** The 8 shared phosphoproteins in the α2-3sia and α2-6sia stimulation with LPS.

Protein Name	UniProt ID	PhosphoSite	α2-3sia		α2-6sia	
			Fold Change	P value	Fold Change	P value
**RGS14**	O43566	Ser288	-2.06	0.0087	2.27	0.0146
**EEF1D**	P29692	Ser133	3.03	0.0330	4.34	0.0180
**SH3KBP1**	Q96B97	Ser587	2.51	0.0119	2.68	0.0493
**EPS15**	P42566	Ser796	-2.23	0.0302	-2.44	0.0231
**SERBP1**	Q8NC51	Ser203	-2.12	0.0182	-2.68	0.0048
**CD200R1**	Q8TD46	Ser297	-5.65	0.0005	-2.58	0.0447
**PXN**	P49023	Ser126	3.25	0.0179	3.04	0.0390
**DDB2**	Q92466	Ser24	-2.72	0.0078	-3.09	0.0133

The overlapping proteins are presented with their protein ID and phosphorylation status upon stimulation. Apart from RGS14, the phosphorylation was similarly affected by the different stimulation conditions. No functional connectivity between these proteins was found.

Despite the large amount of functionally connected nodes, the biological processes affected were difficult to predict. Therefore, we applied gene ontology analysis using the Cytoscape plug-in tool ClueGO to classify in which biological processes the 109 phosphoproteins play a role (37). We continued only with the α2-3sia plus LPS stimulated condition, as the higher number of significantly altered proteins generated a more interconnected GO network compared to the α2-3sia only, α2-6sia only, or α2-6sia with LPS stimulations (**Sl. Figure 2B, 3**). The proteins were annotated to 36 different GO terms and organized in groups. Seven groups were found, including regulation of proliferation, growth hormone response, RNA regulation, growth factor response, organelle and podosome assembly, and SMAD protein signaling (**Figures 3A, B**). The proteins involved with each GO term and groups can be found in **Sl. Table 5**. A smaller cluster was found involving the regulation of IL-12 (**Figure 3A**), which could result in the alterations on the IL-10:IL-12 axis (**Figure 1B**). STAT3 was annotated to each of the GO terms within the IL-12 group. Pathview analysis of the JAK-STAT signaling pathway revealed that other proteins in this specific pathway were affected by α2-3sia and LPS stimulation, including the STAT proteins themselves, SOS, mTOR, CBP, and PIAS (**Figure 3C**) (38). Furthermore, HNRNPA2B1 within the purple IL-12 regulatory group (**Figure 3A** and **Sl. Table 5**) has been associated with the JAK-STAT signaling pathway in T cells, where stimulation with IL-12 resulted in decreased expression of HNRNPA2B1 through STAT signaling (39). The JAK-STAT signaling pathway is therefore an interesting lead to study the mechanism behind the downregulation of IL-12 secretion in DCs upon encounter of α2-3sia and LPS.

### Altered Kinase Signatures After α2-3 Sialic Acid Binding in the Presence of LPS

**Figure 4:**
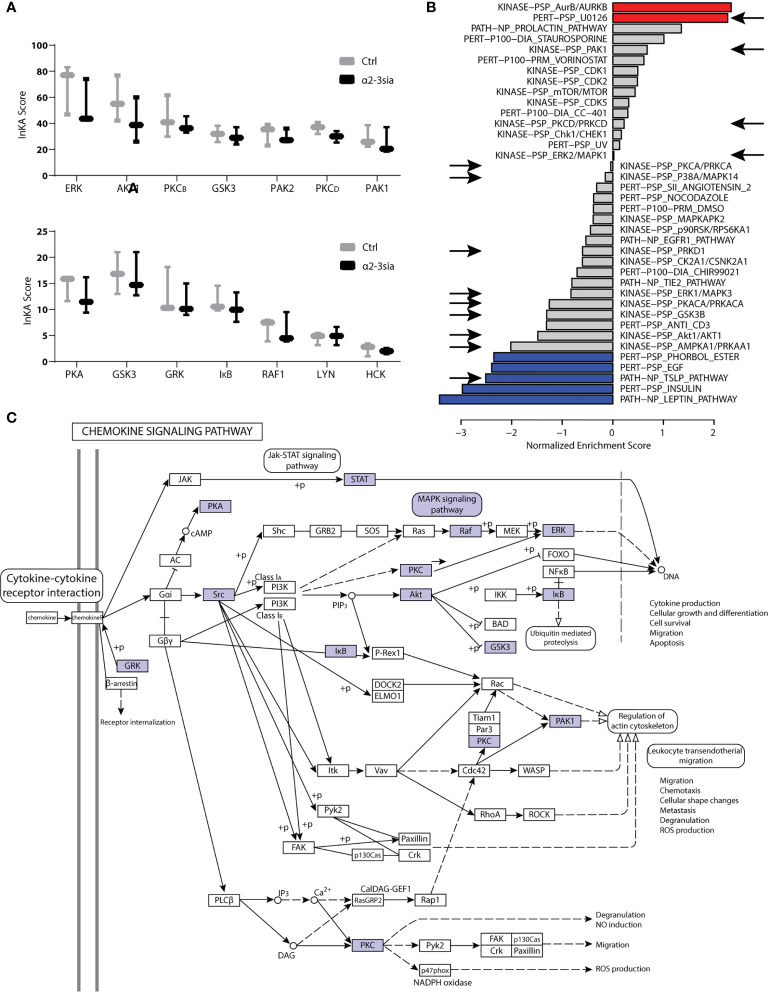
Kinase activity in α2-3sia and LPS stimulation conditions. **(A)** INKA analysis of α2-3sia stimulated moDCs compared to control stimulation all in presence of LPS shows decreased scoring of kinases ERK, AKT1, PKCB, GSK3, PKCD, PAK1, PKA, GSK3, GRK, IκB, and RAF1. **(B)** PTMsigDB signature scoring after stimulation with α2-3sia and LPS is divided into three categories (perturbations, kinases and signatures of molecular pathways). Particularly the red and blue signatures are significantly altered after the stimulation. The signatures appointed by the arrows were also affected in the INKA analysis. **(C)** The affected kinase signatures were involved in the chemokine signaling pathway, indicated by the blue colored kinases.

**Figure 5:**
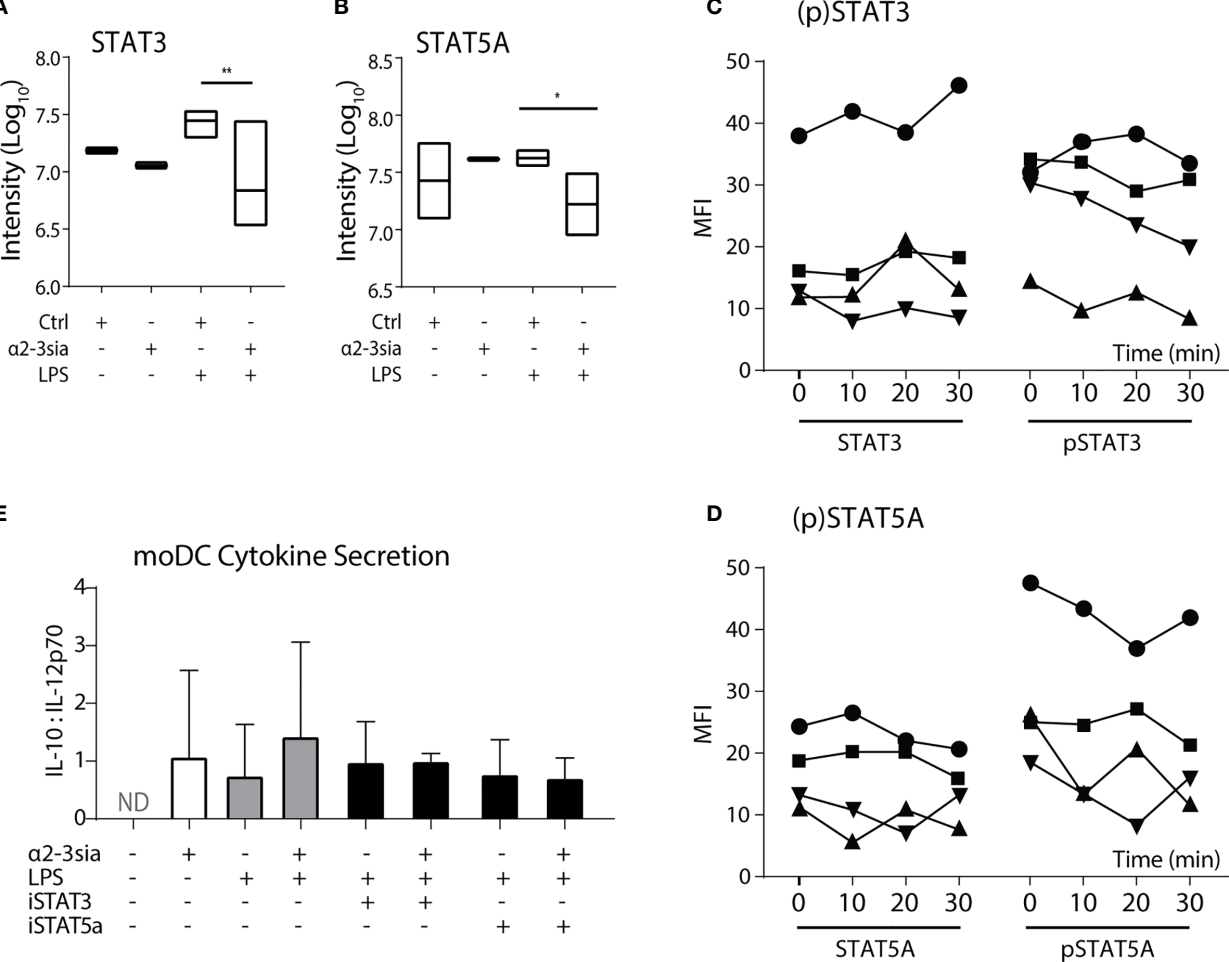
STAT5A phosphorylation is decreased after α2-3 sialic acid binding. **(A)** The phosphoproteomic results (Log10 normalized intensity) of STAT3 phosphorylation after stimulation with the glycodendrimers with or without LPS stimulation. A significant decrease was seen after stimulation with α2-3sia in presence of LPS. **p < 0.01, n=3. **(B)** The phosphoproteomic results (Log10 normalized counts) of STAT5A phosphorylation after stimulation with the glycodendrimers with or without LPS stimulation. A significant decrease was seen after stimulation with α2-3sia stimulation in presence of LPS. *p < 0.05, n=3. **(C)** Flow cytometric quantification of (phosphorylated) STAT3 proteins. A trend towards decreased STAT3 phosphorylation is seen over time. **(D)** Flow cytometric quantification of (phosphorylated) STAT5A proteins of four different individuals. A trend towards decreased STAT5A phosphorylation is seen over time. **(E)** The IL10:IL-12 ratio after overnight stimulation with either of the STAT inhibitors resulted in a similar level as the α2-3sia dendrimer stimulation. Dual stimulation with the inhibitor and dendrimer demonstrated similar results. Range IL-10 22-1061 pg/mL; IL-12 7-1921 pg/mL.

Kinases are essential to signal transduction. Their phosphorylation activity on proteins directs the protein function and localization (40). Integrative Inferred Kinase Activity (INKA) analysis was applied on the phosphoproteomic data to assess the kinase activity after moDC binding to α2-3sia in presence of LPS. This method integrates four phosphoproteomic analyses of one sample to a scoring system, allowing ranking of the kinase activity and visualization of the kinase-substrate networks (23). Multiple kinases were affected by α2-3sia and LPS stimulation (**Figure 4A** and **Sl. Figure 4**). Particularly the scoring of kinases ERK and AKT1 was lower after stimulation, while an overall decreased trend was seen with the affected kinase signature. Additionally, we performed a phosphoproteomic analysis to validate kinase signature found with the INKA scoring. This allowed evaluation of the kinase signatures after α2-3sia and LPS stimulation through Gene Set Enrichment Analysis using a post-translational modification database (PTMsigDB) with site-specific signature information of perturbations, kinase activities and signaling pathways (**Figure 4B** and **Sl. Figure 5**) (24). The red signature scores indicate a significant positive correlation between the signature and data set, while an anti-correlation is reflected by the blue negative scores. The arrows indicate a shared affected signature with the INKA scoring. A significant positive correlation was found of the signature involving U0126, a highly selective inhibitor of the MEK kinase, implying inhibition of the MAPK/ERK signaling pathway after α2-3sia binding to DCs in presence of LPS (41, 42). Furthermore, a negative correlation of the thymic stromal lymphopoietin (TSLP) signature was found. Activation of DCs by TSLP has been linked to the initiation of T_H_2 responses, and to promote triggering of the JAK-STAT pathway (43, 44). A negative correlation was additionally observed with the Leptin and Insulin pathways, although it was not found by INKA scoring. Interestingly, both signatures are involved in promoting DC maturation and migration (45, 46). The negative correlation would therefore indicate that the maturation process of DCs are negatively affected by α2-3sia stimulation. The phosphoproteomic analysis therefore indicates that DC triggering with α2-3sia and LPS is negatively correlated to DC maturation and the induction of inflammatory T cell responses. The kinases that emerged from the INKA and PTMsigDB analyses were mapped to the chemokine signaling pathway (**Figure 4C**). The kinase activity within the pathway is associated with various processes, such as genetic reprogramming and regulation of the actin cytoskeleton. DC binding of α2-3sia in presence of LPS was able to affect this signaling pathway through several kinases. Furthermore, kinase activity within the MAPK signaling pathway was additionally affected, which could lead to altered dendritic cell proliferation and differentiation (**Sl. Figure 6**). These results therefore imply that α2-3sia binding to moDCs enables a kinase activity pattern through similar pathways as DC triggering with chemokines. Alterations in the MAPK/ERK, and JAK-STAT signaling pathway could therefore contribute to skewing of the DC toward a tolerogenic immune status, by means of the altered IL-10:IL-12 secretion axis.

### JAK-STAT Signaling Pathway Is Affected After α2-3 Sialic Acid Binding in the Presence of LPS

Stimulation with α2-3sia in presence of LPS altered multiple proteins within the JAK-STAT signaling pathway (**Figure 3C**). The STAT proteins have been described as important regulators of DC activity and are involved in DC-mediated T cell skewing (31, 32). In the phosphoproteomic quantification, both the phosphorylation of STAT3 and STAT5A was significantly downregulated (**Figures 5A, B**). The STAT3 phosphorylation on serine-727 was 4.08-fold lower. Hypersialylation of cancer cells and secretion of sialic acids in the tumor microenvironment is a common step in cancer cell progression to facilitate immune escape. In NSCLC, α2-3 sialylation was elevated in total serum and phosphorylation of STAT3 Ser727 (and Tyr705) was also reduced in moDCs upon stimulation with sera of multiple non-small cell lung cancer (NSCLC) patients (47), validating the decrease in STAT3 phosphorylation measured here (48).

Phosphorylation of STAT5A on the Ser780 residue was 2.5-fold lower after 30 minutes of α2-3sia stimulation in the presence of LPS (**Figure 5B**). In contrast to STAT3, an immune suppressive role for STAT5A in dendritic cell signaling has not been described yet. Nevertheless, a critical role is reserved for STAT5 in DCs in the skewing of T_H_2, but not T_H_1-type immune responses (32). Phosphorylation of the Ser780 residue is necessary for translocation of the protein to the nucleus to affect genetic reprogramming of DCs during maturation (49). Analysis of the phosphorylation status through flow cytometric measurement showed only little de-phosphorylation of both the STAT proteins over time compared to control (**Figures 5C, D** and **Sl. Figure 7A**). While both the STAT protein quantities remained relatively similar over time, only a small decrease in fluorescent signal was seen at 30 minutes. Quantification of four donors however, demonstrated decreased phosphorylation of STAT3 at 30 minutes, while the phosphorylation status of STAT5A is variable over time between donors. De-phosphorylation was at its lowest already at 20 minutes for two donors, while the other two donors exhibited the decrease at 30 minutes. Overnight moDCs co-stimulation with LPS and either STAT inhibitor demonstrated an increase of IL-10 secretion (**Sl. Figure 7B**). Nonetheless, inhibition of both STAT proteins minimally affected IL-12 secretion (data not shown), resulting in an unaltered IL10:IL-12 ratio compared to α2-3sia stimulation (**Figure 5E**).

α2-3sia stimulation was able to affect the JAK-STAT signaling within LPS-treated DCs by lowering the phosphorylation status of STAT3 and STAT5A after approximately 30 minutes. STAT3 in DCs has already been proposed as a potential therapeutic target for induction of tolerance (50). Inhibition of this protein attenuates immune responses through IL-10 biased skewing of the IL-10:IL-12 axis and thus less effector T cell development. It is therefore tempting to speculate that moDC binding to α2-3sia also results the increase of IL-10 secretion through de-phosphorylation of STAT3 Ser727. Continued investigation of the DC STAT3 phosphorylation and the effect on the cytokine secretion profiles would therefore be highly relevant for insight regarding the induction of DC-mediated immune suppression. For STAT5 the therapeutic outcome is less straightforward. De-phosphorylation of STAT5A after α2-3sia and LPS stimulation suggests decreased nuclear translocation and inhibition of its transcriptional function. Translocation studies could elucidate STAT5A activity and localization within DCs after α2-3sia engagement. Furthermore, the transcriptional role of STAT5 in naïve T cell skewing is highly relevant for the suppression of the effector T cell response induced by α2-3sia and LPS-treated moDCs. Analysis of the T_H_1/T_H_2 skewing after blocking of both STAT proteins in DCs could additionally establish the validity of the JAK-STAT signaling by α2-3sia and LPS stimulation. The importance of the JAK-STAT signaling in the induction of tolerance could furthermore be elucidated *via* co-stimulation with the α2-3sia dendrimer and other TLR stimuli, such as DAMPs. Triggering of JAK-STAT signaling would indicate a central role of this pathway after α2-3sia recognition. Lastly, underlying glycan moieties to the sialic acid can contribute to alterations in Siglec recognition and the induced signaling (51). Larger sialic acid-harboring saccharides that are present *in situ* are therefore very appealing for further exploration. The use of these glycans could additionally contribute to defining the upstream proteins and receptor of the JAK-STAT pathway to provide insight of the induction of tolerance *via* the sialic acid-Siglec axis *in situ* such as in the case of tumor immune evasion (52).

## Conclusion

DCs possess the extraordinary capacity to elicit an appropriate tailored immune response after recognizing internal or external danger signals. This study set out to explore the early events of dendritic cell immune signaling induced upon α2-3 sialic acid dendrimer binding in presence of LPS. Through analysis of phosphoproteomic and kinase activity we found that α2-3 sialic acid modulates LPS stimulation of DCs. The differences in the phosphoproteome induced were not observed in LPS alone nor α2-3sia alone, implying specific modulation of the TLR4 signaling pathway by α2-3sia. Gene ontology revealed that some of these altered proteins were involved in the regulation of IL-12. The IL-10:IL-12 ratio was indeed increased upon α2-3sia stimulation, implying a significant role for the annotated phosphoproteins. Kinome analysis demonstrated a negative correlation with the TSLP signature, which promotes triggering of the JAK-STAT signaling pathway and initiation of T_H_2 responses. The analysis of the DC kinase activity therefore indicates that α2-3sia and LPS triggering results in a kinome that negatively correlates to the induction of inflammatory T cell responses. We additionally identified a decreased phosphorylation of the STAT3 and STAT5A proteins, and the HNRNPA2B1 protein within 30 minutes after addition of α2-3sia to LPS matured DC, again indicating the involvement of the JAK-STAT pathways. Especially the critical role of DC STAT5 in the naïve T cell skewing away from T_H_1-type immune responses is highly relevant in the α2-3sia-mediated DC suppression of the T effector response. The decrease in STAT phosphorylation was furthermore α2-3sia-specific and could not be observed upon α2-6sia binding.

## Data Availability Statement

All relevant data is contained within the article: The original contributions presented in the study are included in the supplementary files. The mass spectrometry proteomics data have been deposited to the ProteomeXchange Consortium via the PRIDE partner repository with the dataset identifier PXD024443 (http://proteomecentral.proteomexchange.org/cgi/GetDataset).

## Author Contributions

R-JL and AH were involved in all experiments and wrote the manuscript. The dendrimers were synthesized by R-JL and HK. Under the supervision of CJ, R-JL and RG-H executed the phosphoproteomic enrichment and isolation, SP executed the LC-Ms/Ms measurements, and TP and AAH performed the statistical analyses. ER and AZ aided R-JL in the visualization of the data, all supervised by SV and YK. All authors contributed to the article and approved the submitted version.

## Funding

This work was funded by the NWO gravitation program 2013 granted to the Institute for Chemical Immunology (ICI-024.002.009) and the LSH-TKI project DC4Balance (LSHM18056-SGF). Cancer Center Amsterdam is acknowledged for support of the proteomics infrastructure.

## Conflict of Interest

The authors declare that the research was conducted in the absence of any commercial or financial relationships that could be construed as a potential conflict of interest.

## References

[1] Novak N Gros E Bieber T Allam J-P. Human Skin and Oral Mucosal Dendritic Cells as “Good Guys” and “Bad Guys” in Allergic Immune Responses. Clin Exp Immunol (2010) 161(1):28–33. 10.1111/j.1365-2249.2010.04162.x 20408854 PMC2940145

[2] Tang D Kang R Coyne CB Zeh HJ Lotze MT. Pamps and DAMPs: Signal 0s That Spur Autophagy and Immunity. Immunol Rev (2012) 249(1):158–75. 10.1111/j.1600-065X.2012.01146.x PMC3662247 22889221

[3] Varki A. Letter to the Glyco-Forum: Since There are PAMPs and DAMPs, There Must be SAMPs? Glycan “Self-Associated Molecular Patterns” Dampen Innate Immunity, But Pathogens can Mimic Them. Glycobiology (2011) 21(9):1121–4. 10.1093/glycob/cwr087 PMC3150115 21932452

[4] Pereira MS Alves I Vicente M Campar A Silva MC Padrão NA. Glycans as Key Checkpoints of T Cell Activity and Function. Front Immunol Front Media SA (2018). 10.3389/fimmu.2018.02754 PMC6277680 30538706

[5] Johannssen T Lepenies B. Glycan-Based Cell Targeting To Modulate Immune Responses. Trends Biotechnol Elsevier Ltd (2017) 334–46. 10.1016/j.tibtech.2016.10.002 28277249

[6] Macauley MS Crocker PR Paulson JC. Siglec-Mediated Regulation of Immune Cell Function in Disease. Nat Rev Immunol (2014) 14(10):653–66. 10.1038/nri3737 PMC4191907 25234143

[7] Varki A. Sialic Acids in Human Health and Disease. Trends Mol Med (2008) 14(8):351–60. 10.1016/j.molmed.2008.06.002 PMC2553044 18606570

[8] Perdicchio M Ilarregui JM Verstege MI Cornelissen LAM Schetters STT Engels S. Sialic Acid-Modified Antigens Impose Tolerance *Via* Inhibition of T-Cell Proliferation and De Novo Induction of Regulatory T Cells. Proc Natl Acad Sci USA (2016) 113(12):3329–34. 10.1073/pnas.1507706113 PMC4812702 26941238

[9] Bandala-Sanchez E Zhang Y Reinwald S Dromey JA Lee B-H Qian J. Cell Regulation Mediated by Interaction of Soluble CD52 With the Inhibitory Receptor Siglec-10. Nat Immunol (2013) 14(7):741–8. 10.1038/ni.2610 23685786

[10] O’Reilly MK Paulson JC. Siglecs as Targets for Therapy in Immune-Cell-Mediated Disease. Trends Pharmacol Sci (2009) 30(5):240–8. 10.1016/j.tips.2009.02.005 PMC2830709 19359050

[11] Rodrigues E Macauley MS. Hypersialylation in Cancer: Modulation of Inflammation and Therapeutic Opportunities. Cancers (Basel) (2018) 10(6). 10.3390/cancers10060207 PMC6025361 29912148

[12] Büll C Boltje TJ van Dinther EAW Peters T de Graaf AMA Leusen JHW. Targeted Delivery of a Sialic Acid-Blocking Glycomimetic to Cancer Cells Inhibits Metastatic Spread. ACS Nano (2015) 9(1):733–45. 10.1021/nn5061964 25575241

[13] Xiao H Woods EC Vukojicic P Bertozzi CR. Precision Glycocalyx Editing as Strategy for Cancer Immunotherapy. PNAS (2016) 113(37):10304–9. 10.1073/pnas.1608069113 PMC5027407 27551071

[14] Severi E Hood DW Thomas GH. Sialic Acid Utilization by Bacterial Pathogens. Microbiology (2007) 153(9):2817–22. 10.1099/mic.0.2007/009480-0 17768226

[15] Chang Y-C Olson J Beasley FC Tung C Zhang J Crocker PR. Group B Streptococcus Engages an Inhibitory Siglec Through Sialic Acid Mimicry to Blunt Innate Immune and Inflammatory Responses In Vivo. PloS Pathog (2014) 10(1):e1003846. 10.1371/journal.ppat.1003846 24391502 PMC3879367

[16] Uchiyama S Sun J Fukahori K Ando N Wu M Schwarz F. Dual Actions of Group B Streptococcus Capsular Sialic Acid Provide Resistance to Platelet-Mediated Antimicrobial Killing. Proc Natl Acad Sci USA (2019) 116(15):7465–70. 10.1073/pnas.1815572116 PMC6462088 30910970

[17] Ritchie ME Phipson B Wu D Hu Y Law CW Shi W. Limma Powers Differential Expression Analyses for RNA-sequencing and Microarray Studies. Nucleic Acids Res (2015) 43(47):e47. 10.1093/nar/gkv007 25605792 PMC4402510

[18] Piersma SR Knol JC de Reus I Labots M Sampadi BK Pham TV. Feasibility of Label-Free Phosphoproteomics and Application to Base-Line Signaling of Colorectal Cancer Cell Lines. J Proteomics (2015) 127:247–58. 10.1016/J.JPROT.2015.03.019 25841592

[19] van der Mijn JC Labots M Piersma SR Pham TV Knol JC Broxterman HJ. Evaluation of Different Phospho-Tyrosine Antibodies for Label-Free Phosphoproteomics. J Proteomics (2015) 127(Pt B):259–63. 10.1016/j.jprot.2015.04.006 25890253

[20] Szklarczyk D Gable AL Lyon D Junge A Wyder S Huerta-Cepas J. String V11: Protein-Protein Association Networks With Increased Coverage, Supporting Functional Discovery in Genome-Wide Experimental Datasets. Nucleic Acids Res (2019) 47(D1):D607–13. 10.1093/nar/gky1131 PMC6323986 30476243

[21] Shannon P Markiel A Ozier O Baliga NS Wang JT Ramage D. Cytoscape: A Software Environment for Integrated Models of Biomolecular Interaction Networks. Genome Res (2003) 13(11):2498–504. 10.1101/gr.1239303 PMC403769 14597658

[22] Luo W Brouwer C. Pathview: An R/Bioconductor Package for Pathway-Based Data Integration and Visualization. Bioinformatics (2013) 29(14):1830–1. 10.1093/bioinformatics/btt285 PMC3702256 23740750

[23] Beekhof R Alphen C Henneman AA Knol JC Pham TV Rolfs F. INKA, an Integrative Data Analysis Pipeline for Phosphoproteomic Inference of Active Kinases. Mol Syst Biol (2019) 15(5). 10.15252/msb.20198981 PMC6533557 31126969

[24] Krug K Mertins P Zhang B Hornbeck P Raju R Ahmad R. A Curated Resource for Phosphosite-Specific Signature Analysis. Mol Cell Proteomics (2019) 18(3):576–93. 10.1074/mcp.TIR118.000943 PMC6398202 30563849

[25] García-Vallejo JJ Ambrosini M Overbeek A van Riel WE Bloem K Unger WWJ. Multivalent Glycopeptide Dendrimers for the Targeted Delivery of Antigens to Dendritic Cells. Mol Immunol (2013) 53(4):387–97. 10.1016/J.MOLIMM.2012.09.012 23103377

[26] Chaffin DO Mentele LM Rubens CE. Sialylation of Group B Streptococcal Capsular Polysaccharide Is Mediated by CpsK and Is Required for Optimal Capsule Polymerization and Expression. J Bacteriol (2005) 187(13):4615–26. 10.1128/JB.187.13.4615-4626.2005 PMC1151781 15968073

[27] Qi H Denning TL Soong L. Differential Induction of Interleukin-10 and Interleukin-12 in Dendritic Cells by Microbial Toll-Like Receptor Activators and Skweing of T-Cell Cytokine Profiles. Infect Immun (2003) 71(6):3337–42. 10.1128/IAI.71.6.3337-3342.2003 PMC155753 12761116

[28] Wojcechowskyj JA Didigu CA Lee JY Parrish NF Sinha R Hahn BH. Quantitative Phosphoproteomics Reveals Extensive Cellular Reprogramming During HIV-1 Entry. Cell Host Microbe (2013) 13(5):613–23. 10.1016/j.chom.2013.04.011 PMC4104530 23684312

[29] Villarroya-Beltri C Gutiérrez-Vázquez C Sánchez-Cabo F Pérez-Hernández D Vázquez J Martin-Cofreces N. Sumoylated HnRNPA2B1 Controls the Sorting of MiRNAs Into Exosomes Through Binding to Specific Motifs. Nat Commun (2013) 4:2980. 10.1038/ncomms3980 24356509 PMC3905700

[30] McCracken S Longman D Marcon E Moens P Downey M Nickerson JA. Proteomic Analysis of SRm160-Containing Complexes Reveals a Conserved Association With Cohesin. J Biol Chem (2005) 280(51):42227–36. 10.1074/jbc.M507410200 16159877

[31] Melillo JA Song L Bhagat G Blazquez AB Plumlee CR Lee C. Dendritic Cell (Dc)-Specific Targeting Reveals Stat3 as a Negative Regulator of DC Function. J Immunol (2010) 184(5):2638–45. 10.4049/jimmunol.0902960 PMC3099405 20124100

[32] Bell BD Kitajima M Larson RP Stoklasek TA Dang K Sakamoto K. The Transcription Factor STAT5 is Critical in Dendritic Cells for the Development of TH2 But Not Th1 Responses. Nat Immunol (2013) 14(4):364–71. 10.1038/ni.2541 PMC4161284 23435120

[33] Shi G-X Harrison K Han S-B Moratz C Kehrl JH Piemonti L. Toll-Like Receptor Signaling Alters the Expression of Regulator of G Protein Signaling Proteins in Dendritic Cells: Implications for G Protein-Coupled Receptor Signaling. J Immunol (2004) 172(9):5175–84. 10.4049/jimmunol.172.9.5175 15100254

[34] Braun MC Kelsall BL. Regulation of Interleukin-12 Production ByG-Protein-Coupled Receptors. Microbes Infect (2001) 3(2):99–107. 10.1016/S1286-4579(00)01357-5 11251296

[35] Lock K Zhang J Lu J Lee SH Crocker PR. Expression of CD33-related Siglecs on Human Mononuclear Phagocytes, Monocyte-Derived Dendritic Cells and Plasmacytoid Dendritic Cells. Immunobiology (2004) 209(1-2):199–207. 10.1016/j.imbio.2004.04.007 15481154

[36] Crocker PR Paulson JC Varki A. Siglecs and Their Roles in the Immune System. Nat Rev Immunol (2007) 7(4):255–66. 10.1038/nri2056 17380156

[37] Bindea G Mlecnik B Hackl H Charoentong P Tosolini M Kirilovsky A. Cluego: A Cytoscape Plug-in to Decipher Functionally Grouped Gene Ontology and Pathway Annotation Networks. Bioinformatics (2009) 25(8):1091–3. 10.1093/bioinformatics/btp101 PMC2666812 19237447

[38] Luo W Pant G Bhavnasi YK Blanchard SG Brouwer C. Pathview Web: User Friendly Pathway Visualization and Data Integration. Nucleic Acids Res (2017) 45(W1):W501–8. 10.1093/nar/gkx372 PMC5570256 28482075

[39] Rosengren AT Nyman TA Lahesmaa R. Proteome Profiling of Interleukin-12 Treated Human T Helper Cells. Proteomics (2005) 5(12):3137–41. 10.1002/pmic.200401151 16038020

[40] Hardie DG. Roles of Protein Kinases and Phosphatases in Signal Transduction. Symp Soc Exp Biol (1990) 44:241–55. 1966636

[41] Duan W Chan JHP Wong CH Leung BP Wong WSF. Anti-Inflammatory Effects of Mitogen-Activated Protein Kinase Kinase Inhibitor U0126 in an Asthma Mouse Model. J Immunol (2004) 172(11):7053–9. 10.4049/jimmunol.172.11.7053 15153527

[42] Marampon F Bossi G Ciccarelli C Di Rocco A Sacchi A Pestell RG. Mek/Erk Inhibitor U0126 Affects in Vitro and in Vivo Growth of Embryonal Rhabdomyosarcoma. Mol Cancer Ther (2009) 8(3):543–51. 10.1158/1535-7163.MCT-08-0570 19258428

[43] Ito T Wang YH Duramad O Hori T Delespesse GJ Watanabe N. Tslp-Activated Dendritic Cells Induce an Inflammatory T Helper Type 2 Cell Response Through OX40 Ligand. J Exp Med (2005) 202(9):1213–23. 10.1084/jem.20051135 PMC2213234 16275760

[44] Shi Z Jiang W Wang M Wang X Li X Chen X. Inhibition of JAK/STAT Pathway Restrains Tslp-Activated Dendritic Cells Mediated Inflammatory T Helper Type 2 Cell Response in Allergic Rhinitis. Mol Cell Biochem (2017) 430(1–2):161–9. 10.1007/s11010-017-2963-7 28214951

[45] Al-Hassi HO Bernardo D Murugananthan AU Mann ER English NR Jones A. A Mechanistic Role for Leptin in Human Dendritic Cell Migration: Differences Between Ileum and Colon in Health and Crohn’s Disease. Mucosal Immunol (2013) 6(4):751–61. 10.1038/mi.2012.113 PMC3684777 23168838

[46] Lu H Huang D Yao K Li C Chang S Dai Y. Insulin Enhances Dendritic Cell Maturation and Scaventer Receptor-Mediated Uptake of Oxidised Low-Density Lipoprotein. J Diabetes Complications (2015) 29(4):465–71. 10.1016/j.jdiacomp.2015.03.005 25813675

[47] Li R Fang F Jiang M Wang C Ma J Kang W. STAT3 and NF-Kb Are Simultaneously Suppressed in Dendritic Cells in Lung Cancer. Sci Rep (2017) 7(1):45395. 10.1038/srep45395 28350008 PMC5368983

[48] Ferens-Sieczkowska M Kratz E Kossowska B Passowicz-Muszyńska E Jankowska R. Comparison of Haptoglobin and Alpha1-Acid Glycoprotein Glycosylation in the Sera of Small Cell and Non-Small Cell Lung Cancer Patients. Postepy Hig Med Dosw (2013) 67:828–36. 10.5604/17322693.1061788 24018448

[49] Berger A Hoelbl-Kovacic A Bourgeais J Hoefling L Warsch W Grundschober E. Pak-Dependent STAT5 Serine Phosphorylation is Required for BCR-ABL-Induced Leukemogenesis. Leukemia (2014) 28(3):629–41. 10.1038/leu.2013.351 PMC3948164 24263804

[50] Barton BE. Stat3: A Potential Therapeutic Target in Dendritic Cells for the Induction of Transplant Tolerance. Expert Opin Ther Targets (2006) 10(3):459–70. 10.1517/14728222.10.3.459 16706685

[51] Alphey MS Attrill H Crocker PR van Aalten DM. High Resolution Crystal Structures of Siglec-7. Insights Into Ligand Specificity in the Siglec Family. J Biol Chem (2003) 31 278(5):3372–7. 10.1074/jbc.M210602200 12438315

[52] Perdicchio M Cornelissen LAM Streng-Ouwehand I Engels S Verstege MI Boon L. Tumor Sialylation Impedes T Cell Mediated Anti-Tumor Responses While Promoting Tumor Associated-Regulatory T Cells. Oncotarget (2016) 7(8):8771–82. 10.18632/oncotarget.6822 PMC4891003 26741508

