# Supplementary material for: Quantitative Phosphoproteomic Analysis Reveals Dendritic Cell- Specific STAT Signaling After α2-3–Linked Sialic Acid Ligand Binding
Source: Front Immunol. 2021 Apr 22;12:673454. doi: 10.3389/fimmu.2021.673454 (PMC8100677; doi:10.3389/fimmu.2021.673454)
Supplement: Supplementary file 1 [file DataSheet_1.docx]

Supplementary Material

# Supplementary Data


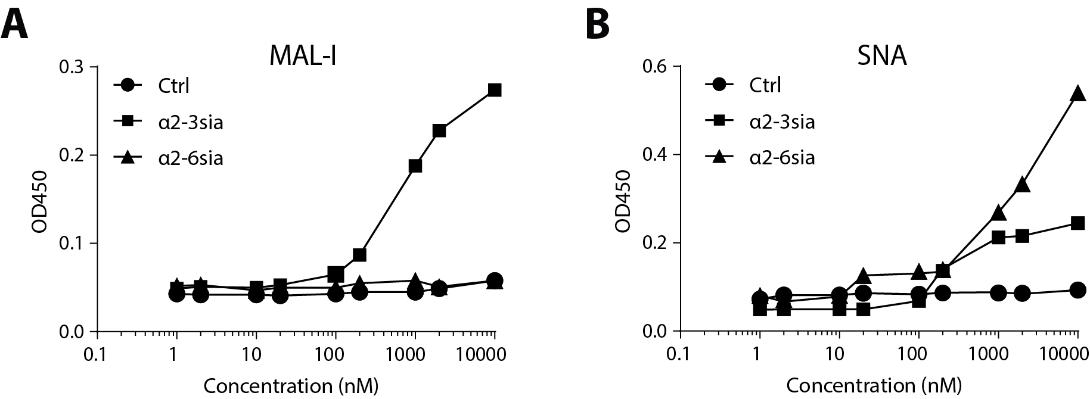


**Sl.Figure 1 | Validation of the sialic acid- glycodendrimers.** (A) Concentration-dependent binding of the glycodendrimers to the α2-3 sialic acid-specific *Maackie* *amurensis* lectin I (MAL-I) in an ELISA assay demonstrates recognition of the α2-3sia dendrimer only. (B) Concentration-dependent binding of the glycodendrimers to *Sambucus* *Nigra* lectin (SNA) in an ELISA assay demonstrates recognition of the α2-6sia dendrimer. SNA is able to bind α2-3 sialic acid-linked saccharides to a lesser degree.


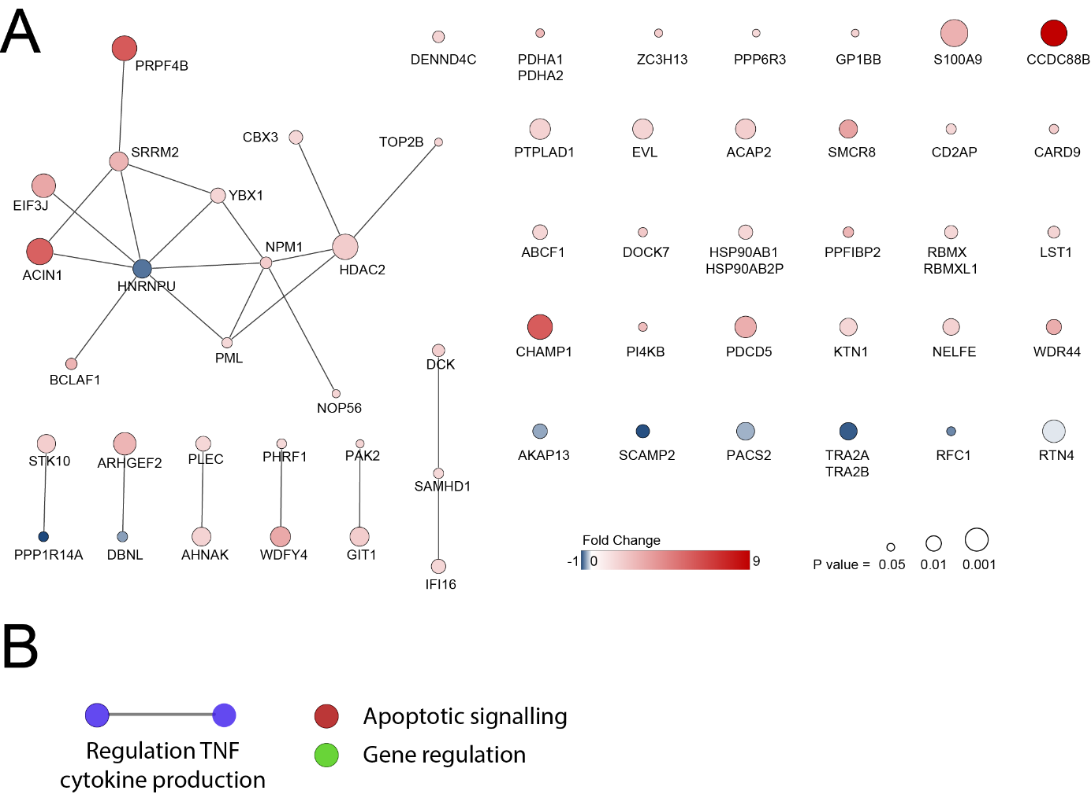


**Sl.Figure 2 | Interconnectivity of the α2-3sia altered proteins.** (A) STRING analysis reveal a network of 13 phosphoproteins significantly affected by α2-3 sialic acid binding without secondary LPS stimulation. (B) GO term enrichment analysis through ClueGO of the α2-3sia altered proteins.


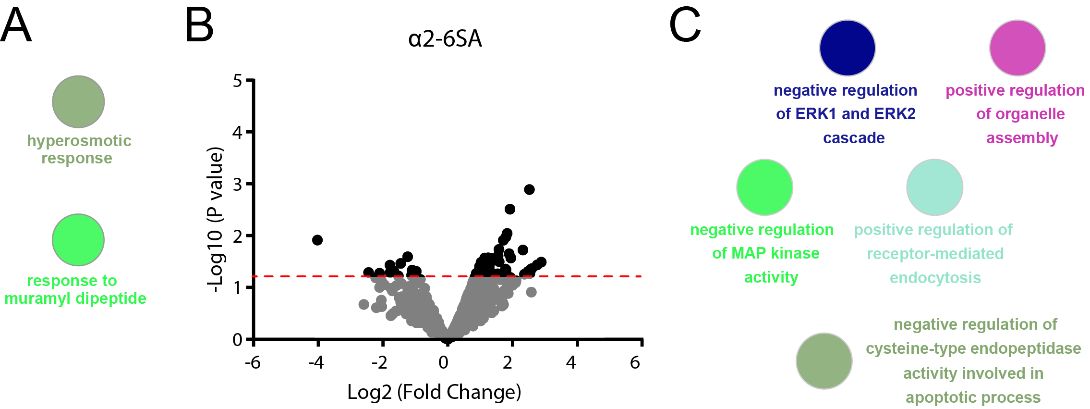


**Sl.Figure 3 | Interconnectivity of the α2-3sia and α2-6sia altered proteins.** (A) GO term enrichment analysis through ClueGO of the α2-3sia altered proteins without of LPS mapped one small network. (B) The Volcano plot demonstrates modified phosphoprotein expression after stimulation with the α2-6 sialic acid dendrimer only. The data above de red dotted line represent all the significantly alter phosphoproteins over three donors. The –Log10 of the average p values, calculated with a limma test, is presented against the Log2 fold in fold change. (C) GO term enrichment analysis through ClueGO of the α2-6sia altered proteins without of LPS did not map to any networks.


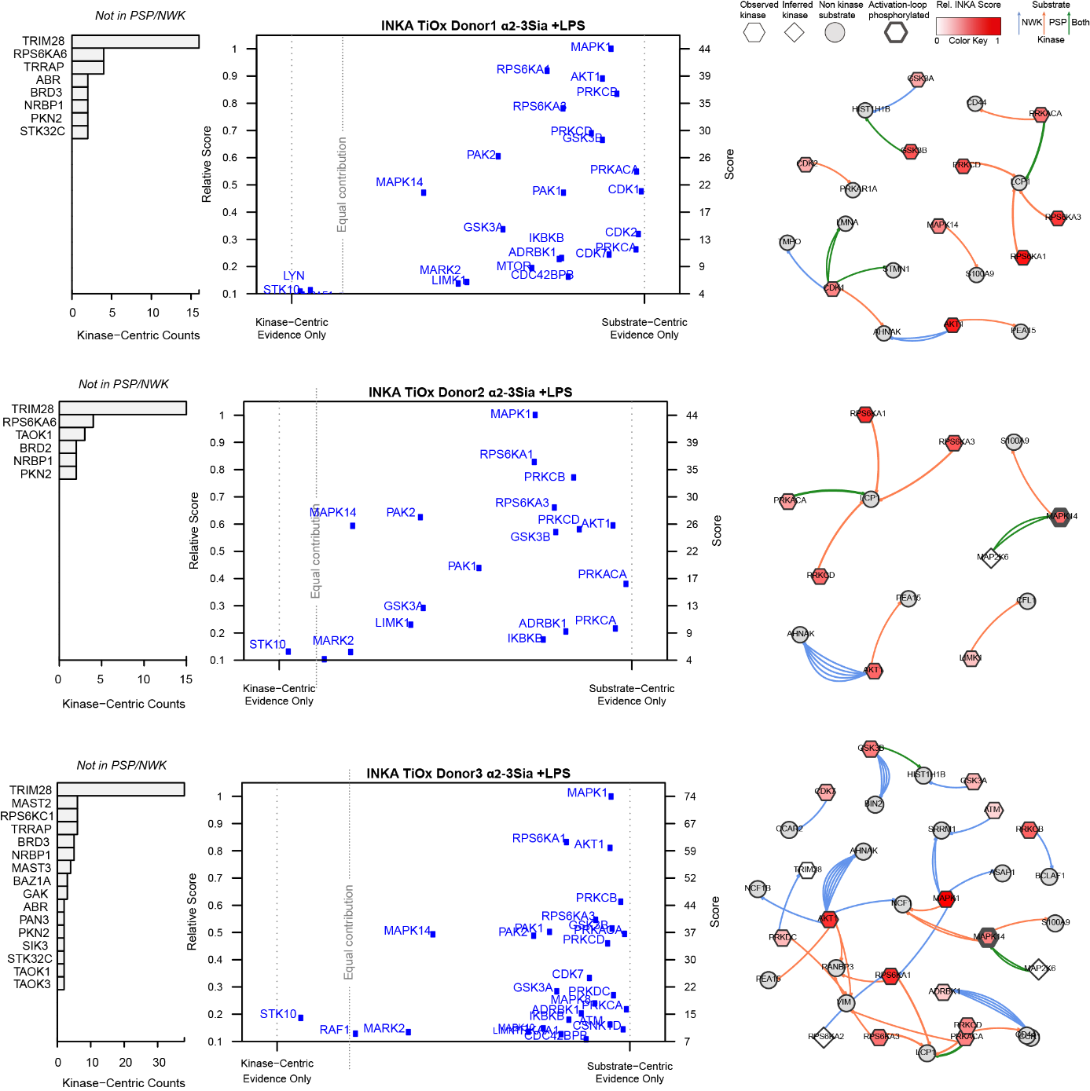


**Sl.Figure 4 | INKA plots and kinase-substrate relation networks.** The INKA plots of the three donors after stimulation with α2-3sia and LPS stimulation. The left graph visualizes the kinases not covered by PhosphoSitePlus (PSP) and NetworKIN (NWK) in the middle plot. The INKA TiOx plots the kinases on INKA scoring (y-axis) and the balance of kinase- or substrate-centric evidence. The righter networks visualize the kinases and substrates within a single sample.


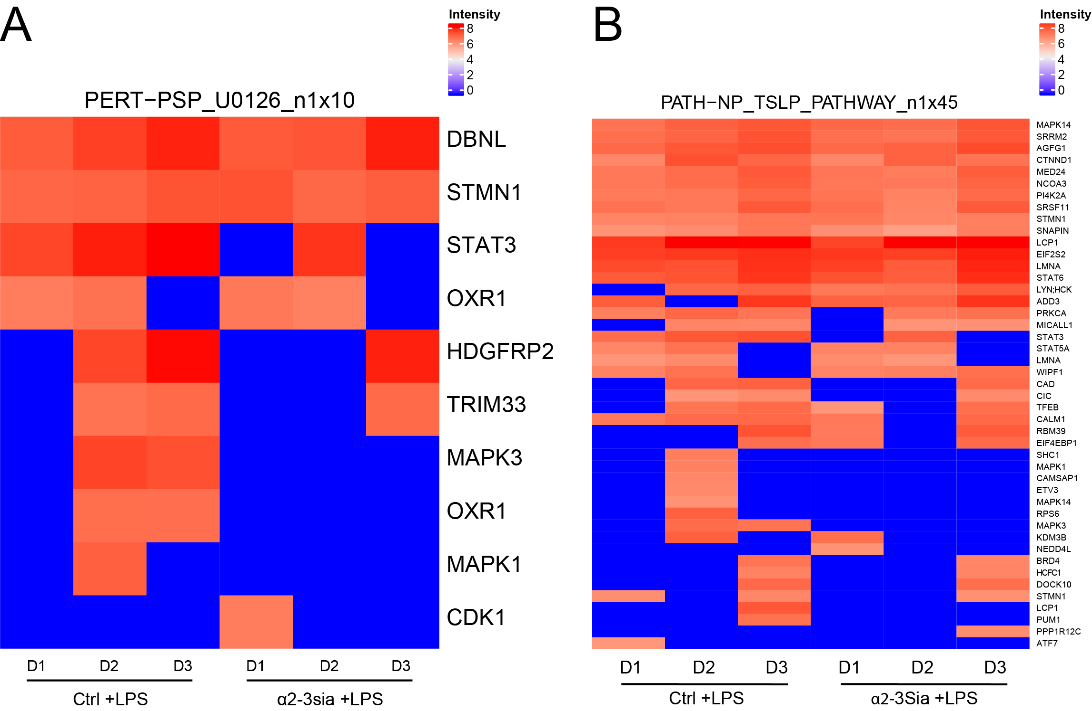


**Sl.Figure 5 | α2-3sia-specific kinase signatures in presence of LPS.** (A) Heatmap depicting the kinase signature of U0126. (B) Heatmap depicting the kinase signature of the TSLP pathway.


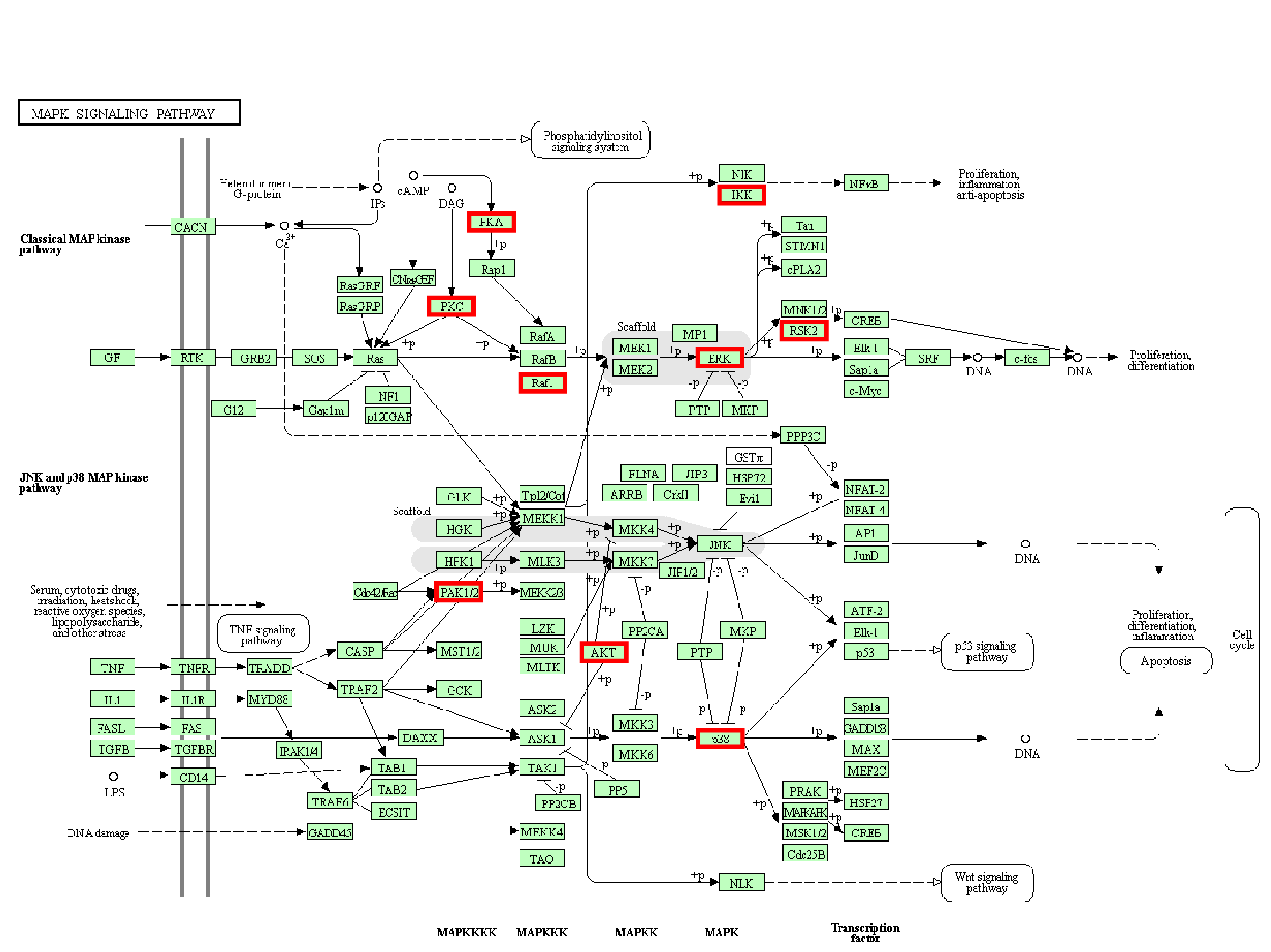


**Sl.Figure 6 | Model of MAPK signaling pathway.** The affected kinase signatures that were affected in the MAPK signaling pathway are indicated by the red colored rectangles.


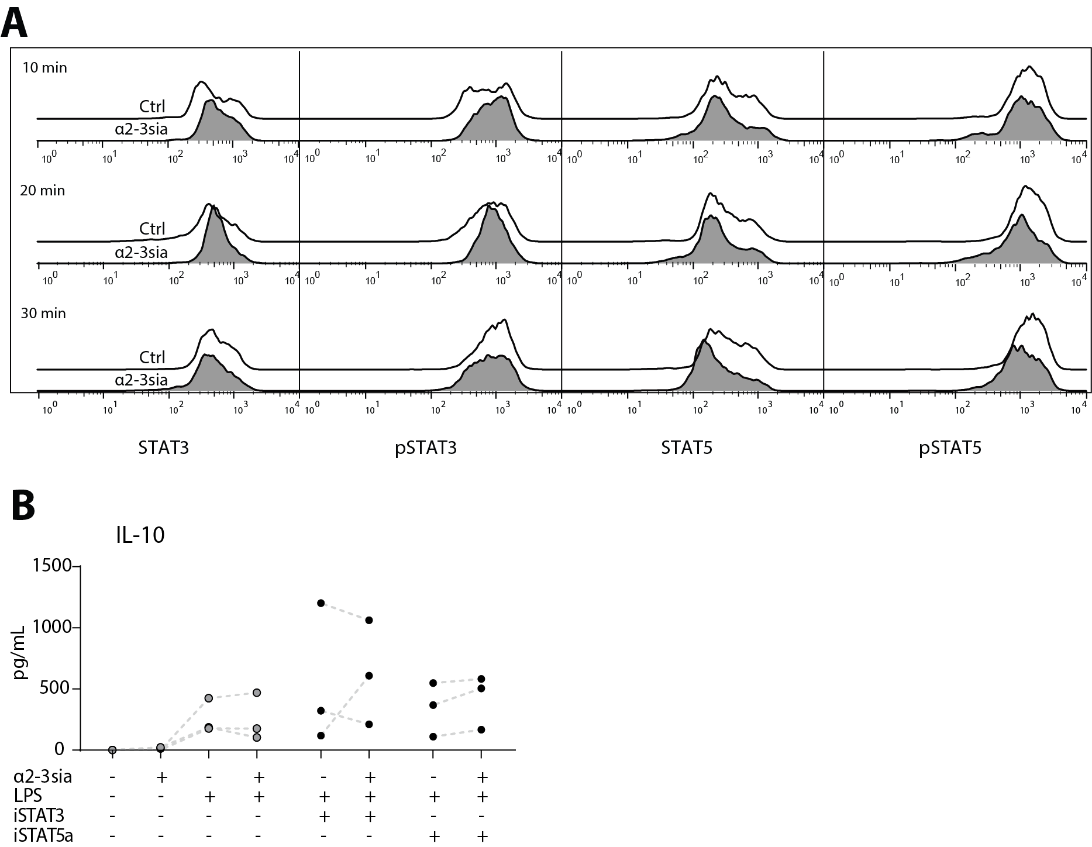


**Sl.Figure 7 | STAT5A phosphorylation is decreased after α2-3 sialic acid binding.** (A) Flow cytometric analysis of the STAT and phosphorylated STAT proteins in grey, compared to control in white. A minimal decrease in signal is seen at 30 minutes of the pSTAT5A. One donor is depicted as representative of four individuals. (B) After overnight stimulation of the moDCs with the dendrimers and the STAT inhibitors, IL-10 was quantified in the supernatant through an ELISA assay. Stimulation with either of the STAT inhibitors increased the IL-10 secretion, which was enhanced with additional α2-3sia stimulation.

**Sl.Table 1 | Significantly altered phosphoproteins after α2-3sia stimulation.** The red highlighted proteins are discussed in this paper.

| Gene Name | UNIPROT ID | Positions within proteins | | FC | P Value |
| --- | --- | --- | --- | --- | --- |
| EIF3J | O75822, -2 -3 | S | 11 | 3.409 | 0.001 |
| SCAMP2 | O15127 | S | 319 | -0.643 | 0.003 |
| SRRM2 | Q9UQ35, -2 | S | 1403 | 2.285 | 0.003 |
| DBNL | Q9UJU6, -2, -3, -4, -5, -6 | S | 166;269;175;270;278;221 | -0.352 | 0.005 |
| PACS2 | Q86VP3, -2, -3, -4 | S | 378;453;454 | -0.282 | 0.005 |
| STK10 | O94804 | S | 448 | 1.891 | 0.006 |
| PML | P29590, -2, -3, -4, -5, -8, -9, -11, -12, -13 | S | 518;470 | 1.530 | 0.006 |
| SAMHD1 | Q9Y3Z3, -2, -3, -4 | S | 18 | 1.535 | 0.008 |
| NPM1 | P06748, -2, -3 | S | 125 | 1.932 | 0.008 |
| BCLAF1 | Q9NYF8, -2, -3 | S | 510;512 | 2.753 | 0.009 |
| RBMX | P38159, -2 | S | 195;208 | 1.552 | 0.012 |
| KTN1 | Q86UP2, -2, -3, -4 | S | 75 | 1.610 | 0.012 |
| ARHGEF2 | Q92974, -2, -3 | S | 94;121 | 2.871 | 0.014 |
| PHRF1 | Q9P1Y6, -2, -3 | S | 914;915 | 1.489 | 0.016 |
| ZC3H13 | Q5T200, -2 | S | 986 | 1.940 | 0.018 |
| PPP1R14A | Q96A00, -2 | S | 26 | -0.668 | 0.018 |
| BCLAF1 | Q9NYF8, -2, -3 | S | 383;385 | 1.706 | 0.018 |
| PLEC | Q15149, -2, -3, -4, -5, -6, -7, -8, -9 | S | 4499;4503;4444;4454;4462;4476;4480;4613;4476 | 1.574 | 0.018 |
| PLEC | Q15149, -2, -3, -4, -5, -6, -7, -8, -9 | S | 4502;4506;4447;4457;4465;4479;4483;4616;4479 | 1.554 | 0.020 |
| GIT1 | Q9Y2X7, -3 | S | 592;601 | 1.939 | 0.021 |
| WDR44 | Q5JSH3, -2, -4 | T | 219;219;194 | 3.208 | 0.021 |
| DENND4C | Q5VZ89, -2, -5, -6 | S | 732;732;208;732 | 1.670 | 0.022 |
| HSP90AB1 | P08238 | S | 255;177 | 1.581 | 0.022 |
| RFC1 | P35251, -2 | S | 69 | -0.436 | 0.022 |
| PPP6R3 | Q5H9R7, -2, -3, -4, -5, 6 | S | 537;588;611;617 | 1.494 | 0.023 |
| EVL | Q9UI08, -2, -3 | S | 306;304;310 | 1.674 | 0.023 |
| NOP56 | O00567 | S | 569 | 1.640 | 0.023 |
| CHAMP1 | Q96JM3 | S | 427 | 6.347 | 0.023 |
| TOP2B | Q02880, -2 | S | 1517;1522 | -0.531 | 0.024 |
| CCDC88B | A6NC98, -4 | S | 1227;1345 | 9.928 | 0.024 |
| AHNAK | Q09666 | S | 210 | 1.705 | 0.027 |
| GP1BB | P13224, -2 | S | 191;396 | 1.765 | 0.030 |
| ABCF1 | Q8NE71, -2 | S | 105 | 1.591 | 0.031 |
| CBX3 | Q13185 | S | 176 | 1.553 | 0.031 |
| SRRM2 | Q9UQ35, -2 | S | 1179 | 2.927 | 0.031 |
| RTN4 | Q9NQC3, -2, -3, -4 | S | 7 | -0.089 | 0.032 |
| SRRM2 | Q9UQ35 | S | 2449;2031 | 2.138 | 0.032 |
| S100A9 | P06702 | T | 113 | 3.021 | 0.032 |
| HDAC2 | Q92769, -3 | S | 364;394 | 1.990 | 0.034 |
| SRRM2 | Q9UQ35 | S | 2100 | 2.302 | 0.035 |
| BCLAF1 | Q9NYF8, -2, -3, -4 | S | 266;268 | 1.866 | 0.035 |
| WDFY4 | Q6ZS81, -4, -5 | S | 1846 | 3.338 | 0.036 |
| YBX1 | P67809 | S | 165 | 1.622 | 0.037 |
| CARD9 | Q9H257, -2 | S | 424 | 1.971 | 0.038 |
| PDCD5 | O14737 | S | 119 | 3.162 | 0.039 |
| SMCR8 | Q8TEV9, -2 | S | 487 | 3.588 | 0.039 |
| NELFE | P18615, -3, -4 | S | 49;56 | -0.633 | 0.040 |
| DOCK7 | Q96N67, -2, -3, -4, -5, -6 | S | 896 | 2.089 | 0.042 |
| TRA2A | Q13595, -3, -4 | S | 264;164;260;159;158 | -0.599 | 0.042 |
| PRPF4B | Q13523 | S | 366 | 6.442 | 0.042 |
| SRRM2 | Q9UQ35 | S | 2032 | 2.079 | 0.042 |
| SRRM2 | Q9UQ35 | S | 2020 | 1.617 | 0.042 |
| ACIN1 | Q9UKV3, -5 | S | 208 | 6.170 | 0.043 |
| HNRNPU | Q00839, -2 | S | 3 | -0.510 | 0.044 |
| CD2AP | Q9Y5K6 | S | 458 | 1.542 | 0.045 |
| AKAP13 | Q12802, -2, -4 | S | 1886;1904;1908 | -0.318 | 0.045 |
| NELFE | P18615, -3, -4 | S | 353;360;323 | 1.770 | 0.045 |
| LST1 | O00453, -10 | S | 18;49 | 1.662 | 0.046 |
| PI4KB | Q9UBF8-2 | S | 294 | 2.605 | 0.046 |
| PPFIBP2 | Q8ND30, -2, -3 | S | 186;217;329 | 2.848 | 0.047 |
| IFI16 | Q16666, -2, -3 | S | 153 | 1.614 | 0.047 |
| TOP2B | Q02880, -2 | S | 1519;1524 | 1.652 | 0.047 |
| DCK | P27707 | S | 74 | 1.887 | 0.047 |
| HACD3 | Q9P035 | S | 114 | 1.770 | 0.048 |
| PDHA1 | P08559, -2, -3, -4 | S | 262;293;300;331;291 | 2.737 | 0.048 |
| PAK2 | Q13177 | S | 141 | 1.686 | 0.049 |
| ACAP2 | Q15057 | S | 521 | 1.911 | 0.049 |
| BCLAF1 | Q9NYF8, -2, -3, -4 | S | 283;285 | 2.902 | 0.049 |

**Sl.Table 2 | Significantly altered phosphoproteins after α2-3sia with LPS stimulation.** The red highlighted proteins are discussed in this paper. The STAT proteins that are validated in this paper are encircled in bold lining.

| Gene Name | UNIPROT | Positions within proteins | | FC | P value |
| --- | --- | --- | --- | --- | --- |
| HNRNPA2B1 | P22626, -2 | S | 247;259 | -10.204 | 0.000 |
| CD200R1 | Q8TD46, -4 | S | 279;302 | -5.655 | 0.000 |
| RPRD2 | Q5VT52, -2, -3, -5 | S | 556;567;593 | -3.575 | 0.001 |
| SRRM2 | Q9UQ35, -2 | T | 1043 | 9.190 | 0.002 |
| TKT | P29401, -2 | S | 295;303 | -7.568 | 0.003 |
| MADD | Q8WXG6, -2, -3,-4, -5, -6, -7, -8 | S | 770;813 | 7.244 | 0.003 |
| ZFP36 | P26651 | S | 93 | -3.122 | 0.003 |
| PLEC | Q15149, -2, -3, -4, -5, -6, -8, -9 | S | 3162;3166;3107;3117;3125;3139;3143;3276;3139 | 6.793 | 0.003 |
| WDR44 | Q5JSH3, -2, -4 | S | 403;378 | 6.315 | 0.004 |
| STIM1 | Q13586 | S | 519 | -2.841 | 0.005 |
| LRCH4 | O75427 | S | 513 | -5.073 | 0.005 |
| RPS6 | P62753 | S | 235 | -2.167 | 0.006 |
| EPB41L2 | O43491-4 | S | 87 | 5.494 | 0.006 |
| SLCO2B1 | O94956, -2, -3, -4 | S | 93;176;298;320 | -5.442 | 0.006 |
| DLGAP4 | Q9Y2H0, -1, -3 | S | 434;970;973 | -3.955 | 0.007 |
| AHNAK | Q09666 | T | 4100 | -2.325 | 0.007 |
| DDB2 | Q92466, -2, -3, -4, -5 | S | 24 | -2.721 | 0.008 |
| STAT3 | P40763, -2 | S | 726;727 | -4.077 | 0.008 |
| RGS14 | O43566, -4, -5, -6 | S | 288;135;68;135 | -2.062 | 0.009 |
| ARHGAP9 | Q9BRR9, -2, -3, -4, -5 | T | 283;99 | -2.356 | 0.009 |
| SH3KBP1 | Q96B97, -2, -3 | S | 550;587;349 | 2.510 | 0.012 |
| USP9X | Q93008, -1 | S | 2443 | 4.166 | 0.012 |
| AHNAK | Q09666 | S | 5393 | -1.885 | 0.012 |
| SPN | P16150 | S | 355 | -4.132 | 0.012 |
| TFEB | P19484 | S | 109 | -4.125 | 0.012 |
| LSP1 | P33241, -2, -3 | S | 188;316;126 | -4.115 | 0.013 |
| IWS1 | Q96ST2, -2, -3 | S | 377;52;170 | -2.359 | 0.013 |
| FAM126A | Q9BYI3, -2 | S | 415;74 | -2.800 | 0.014 |
| CDC42BPB | Q9Y5S2 | S | 1690 | -2.439 | 0.014 |
| EIF6 | P56537, -2 | S | 224;243 | -3.966 | 0.014 |
| ACIN1 | Q9UKV3, -5 | S | 208;208 | -3.487 | 0.014 |
| BIN2 | Q9UBW5, -2 | S | 240;272 | -2.426 | 0.015 |
| LSP1 | P33241, -2, -3 | S | 177;305;115 | -2.097 | 0.015 |
| MIA3 | Q5JRA6, -2 | S | 727 | -3.741 | 0.017 |
| MARK2 | Q7KZI7, -2, -3, -4, -6, -8, -9, -10, -11, -12, -13, -14, -15, -16 | S | 452;485;453;486 | -2.409 | 0.017 |
| SUZ12 | Q15022 | S | 546 | -2.081 | 0.017 |
| PRKAB1 | Q9Y478 | S | 108 | -3.009 | 0.018 |
| PXN | P49023, -2, -3 | S | 126 | 3.255 | 0.018 |
| SERBP1 | Q8NC51, -2, -3, -4 | S | 203 | -2.119 | 0.018 |
| PCBP1 | Q15365 | S | 189 | -4.358 | 0.018 |
| ARHGAP1 | Q07960 | S | 47 | -2.245 | 0.019 |
| DNAJC5 | Q9H3Z4, -2 | S | 10 | -3.490 | 0.020 |
| PPP1R12A | O14974, -2, -3, -4, -5 | S | 420;507 | -2.150 | 0.020 |
| FOXK1 | P85037, -2 | S | 416;253 | -1.739 | 0.022 |
| PPP1R12A | O14974, -2, -3, -4, -5 | S | 212;299 | -3.408 | 0.022 |
| STAT5A | P42229, -2 | S | 750;780 | -2.534 | 0.022 |
| LSP1 | P33241, -2, -3 | S | 204;332;142 | -1.892 | 0.023 |
| TBC1D9B | Q66K14, -2 | S | 411 | 3.249 | 0.025 |
| ARHGAP9 | Q9BRR9, -3, -4, -5 | S | 456;475;272;291 | -1.783 | 0.026 |
| FMNL1 | O95466, -2, -3 | S | 184 | -1.744 | 0.027 |
| BCLAF1 | Q9NYF8, -2, -3 | S | 383;385 | -2.079 | 0.027 |
| TBC1D5 | Q92609, -2 | S | 554;576 | -1.949 | 0.027 |
| ZFP36 | P26651 | S | 323 | -3.188 | 0.027 |
| UBAP2L | Q14157, -1, -3, -4, -5 | S | 597;604 | -1.830 | 0.028 |
| PRKAB2 | O43741, -2 | S | 102;184 | -1.772 | 0.028 |
| MTDH | Q86UE4 | S | 298 | -5.031 | 0.029 |
| SRRM2 | Q9UQ35 | S | 2118 | -2.015 | 0.029 |
| FLVCR2 | Q9UPI3, -2 | S | 307;512 | -3.087 | 0.030 |
| EPS15 | P42566, -2 | S | 796;482 | -2.228 | 0.030 |
| STMN1 | P16949, -2 | S | 16; | -3.063 | 0.031 |
| BCKDK | O14874, -2, -3 | S | 31 | -1.999 | 0.031 |
| FKBP15 | Q5T1M5, -2 | S | 1161;1151 | -1.735 | 0.031 |
| NDRG3 | Q9UGV2, -2, -3 | S | 242;319;331 | -3.432 | 0.031 |
| ARHGEF11 | O15085, -2 | S | 1458;1498 | -1.994 | 0.032 |
| AHNAK | Q09666 | S | 5749 | -1.655 | 0.032 |
| EEF1D | P29692,-2, -3, -4 | S | 133;499;114;109 | 3.033 | 0.033 |
| PCM1 | Q15154, -2, -3 | S | 65 | -2.015 | 0.033 |
| RBMX | P38159, -2 | S | 339;352 | -1.665 | 0.035 |
| MYO18A | Q92614, -2, -3, -4, -5 | S | 1983;1998;1946;1525;1667 | -2.971 | 0.036 |
| PEA15 | Q15121, -2 | S | 116;137 | -1.758 | 0.037 |
| STK10 | O94804 | S | 448 | 1.632 | 0.037 |
| MAP1S | Q66K74, -2 | S | 703;729 | -2.122 | 0.037 |
| ARHGEF2 | Q92974, -2, -3 | S | 145;172 | -1.971 | 0.037 |
| PRPSAP2 | O60256, -2, -3, -4 | S | 141;227;187 | -1.982 | 0.037 |
| COL19A1 | Q14993 | S | 81 | 1.928 | 0.037 |
| NCOR2 | Q9Y618-2, -3, -4, -5 | S | 1690 | -2.765 | 0.037 |
| MAP3K7 | O43318, -2, -3, -4 | S | 389 | -2.868 | 0.038 |
| HIST1H1C | P16403 | S | 2 | -2.512 | 0.039 |
| LCP1 | P13796 | S | 5 | -1.867 | 0.039 |
| RBM14 | Q96PK6 | S | 280 | -1.678 | 0.039 |
| NMT1 | P30419 | S | 83;3 | -1.910 | 0.039 |
| NCL | P19338 | S | 563 | -1.685 | 0.039 |
| JUN | P05412 | S | 63 | -1.744 | 0.040 |
| LARP4B | Q92615 | S | 601 | -1.620 | 0.041 |
| PI4K2A | Q9BTU6 | S | 5 | -2.786 | 0.042 |
| ATXN2L | Q8WWM7, -3, -4, -5, -6, -8, -9 | S | 558 | -1.877 | 0.042 |
| MAP4K1 | Q92918, -2 | S | 405 | -1.824 | 0.042 |
| MYCBP2 | O75592, -2 | S | 2833 | -2.047 | 0.043 |
| ABL2 | P42684, -2, -3, -4, -5, -6, -7 | S | 781;796;802;817;678;693;699;714 | -1.610 | 0.043 |
| RPL24 | P83731 | S | 86 | -1.974 | 0.043 |
| PRKAR1A | P10644, -2 | S | 77 | -1.880 | 0.043 |
| THRAP3 | Q9Y2W1 | S | 248 | -1.718 | 0.044 |
| ARHGEF2 | Q92974, -2, -3 | S | 617;644;645 | -1.592 | 0.044 |
| LILRB4;LILRB1 | Q8NHJ6, -2, -3, -4 | S | 379;380;381;582;583;583;584 | -1.804 | 0.044 |
| EIF2S2 | P20042 | S | 2 | -1.991 | 0.045 |
| RTN4 | Q9NQC3, -2, -4, -5 | S | 7 | -2.574 | 0.045 |
| NFRKB | Q6P4R8, -2, -3 | S | 1290;1291;1316 | -2.718 | 0.045 |
| MAP1A | P78559, -2 | S | 2106 | -2.323 | 0.045 |
| LARP7 | Q4G0J3, -3 | S | 298;305 | -1.911 | 0.047 |
| SYNRG | Q9UMZ2, -3, -4, -5, -6, -7, -8, -9 | S | 869;952;996;997;1075 | -1.728 | 0.047 |
| EIF4G3 | O43432, -3 | S | 495;501 | -1.925 | 0.047 |
| LILRB4;LILRB1 | Q8NHJ6, -2, -3, -4 | S | 376;377;378;579;580;581 | -1.584 | 0.048 |
| VIM | P08670 | S | 459 | 2.833 | 0.048 |
| SAMHD1 | Q9Y3Z3, -2, -3, -4 | S | 18 | -2.677 | 0.049 |
| SSFA2 | P28290, -2, -3 | S | 584;737 | -1.594 | 0.049 |
| MAP4 | P27816, -2, -6 | S | 358 | -1.676 | 0.049 |

**Sl.Table 3 | Significantly altered phosphoproteins after α2-6sia stimulation.**

| Gene Names | UNIPROT ID | Positions within proteins | | FC | P Value |
| --- | --- | --- | --- | --- | --- |
| AP3B1 | O00203, -3 | S | 227;276 | -0.315 | 0.001 |
| PDCD5 | O14737 | S | 119 | 5.869 | 0.003 |
| RGS14 | O43566, -6 | S | 20 | 5.746 | 0.009 |
| HSP90AA1 | P07900, -2 | S | 263;385 | 3.863 | 0.011 |
| HCLS1 | P14317 | S | 275 | 2.149 | 0.012 |
| PTPN7 | P35236, -2, -3 | S | 143;182;248 | 3.009 | 0.012 |
| HNRNPK | P61978, -2, -3 | S | 284;260 | -0.423 | 0.018 |
| RNF213 | Q63HN8, -4 | S | 1258;1307 | 2.234 | 0.019 |
| BUD13 | Q9BRD0 | S | 325 | 2.436 | 0.022 |
| RANBP3 | Q9H6Z4, -2, -3 | S | 265;328;333 | 1.975 | 0.025 |
| BCLAF1 | Q9NYF8, -2, -3 | S | 510;512 | 2.280 | 0.026 |
| HACD3 | Q9P035 | S | 138;83 | 3.480 | 0.027 |
| HACD3 | Q9P035 | S | 114 | 2.041 | 0.027 |
| EVL | Q9UI08, -2 | S | 371;369 | -0.469 | 0.027 |
| PCYT1B | Q9Y5K3, -2, -3, -4 | S | 297;315 | 2.532 | 0.027 |
| PPP1R18 | Q6NYC8, -2 | T | 113 | 6.054 | 0.030 |
| CFL1 | P23528 | T | 71;88 | 1.980 | 0.031 |
| XRCC6 | P12956, -2 | S | 475;434 | 3.491 | 0.032 |
| LSP1 | P3324, -2, -3 | S | 130;258;68 | 3.564 | 0.032 |
| EPS15 | P42566, -2 | S | 562;248 | 3.720 | 0.033 |
| HDGF | P51858, -2, -3 | S | 132;125;148 | 2.935 | 0.034 |
| HDGF | P51858, -2, -3 | S | 133;126;149 | 2.213 | 0.036 |
| MFAP1 | P55081 | S | 52 | 2.448 | 0.037 |
| CORO7 | P57737, -2, -3, -4 | S | 377;444;462 | 3.284 | 0.037 |
| HNRNPU | Q00839, -2 | S | 3 | -0.512 | 0.037 |
| NCBP1 | Q09161 | S | 22 | 2.375 | 0.039 |
| PAK1 | Q13153, -2 | S | 204 | 5.603 | 0.039 |
| RABL6 | Q3YEC7, -2 | S | 596;597 | 2.364 | 0.039 |
| WDFY4 | Q6ZS81, -4, -5-4 | S | 1846 | 3.449 | 0.040 |
| ZC3H18 | Q86VM9, -2 | S | 532;500 | -0.062 | 0.043 |
| PPFIBP2 | Q8ND30, -2, -3 | S | 219;250;362 | -0.367 | 0.043 |
| TOM1 | O60784, -2, -3, -4 | S | 416;429;461;462 | 7.425 | 0.043 |
| ARHGEF2 | Q92974, -2, -3 | S | 94;121 | 5.002 | 0.043 |
| LILRB3 | O75022, -2, -3 | S | 502;519 | 1.981 | 0.044 |
| HN1 | Q9UK76, -2, -3 | S | 41;87 | 2.325 | 0.045 |
| RALY | Q9UKM9, -2 | S | 272;288 | -0.291 | 0.046 |
| SRRM2 | Q9UQ35, -2 | S | 1403 | 3.796 | 0.046 |
| SRRM2 | Q9UQ35 | S | 2032 | 2.996 | 0.048 |
| AKAP2 | Q9Y2D5, -4, -5, -6 | S | 152;241;383 | 6.780 | 0.049 |
| SH3BP1 | Q9Y3L3 | S | 585 | 2.425 | 0.049 |

**Sl.Table 4 | Significantly altered phosphoproteins after α2-6sia with LPS stimulation.**

| Gene Names | Proteins | Positions within proteins | | FC | P value |
| --- | --- | --- | --- | --- | --- |
| KIAA1598 | A0MZ66, -3, -4, -5, -6, -7, -8 | S | 407;437;467;55 | -4.049 | 0.012 |
| ZNRF2 | Q8NHG8 | S | 19 | -12.411 | 0.001 |
| FIP1L1 | Q6UN15, -3, -5 | S | 486;492;418 | -7.153 | 0.001 |
| NOP56 | O00567 | S | 519 | 3.116 | 0.003 |
| RAF1 | P04049, -2 | S | 289;309 | -17.602 | 0.004 |
| EPS15 | P42566, -2 | S | 796;482 | -15.557 | 0.004 |
| STAT5B | P51692 | S | 128 | 3.353 | 0.005 |
| SCAMP2 | O15127 | S | 319 | -2.677 | 0.005 |
| FNBP4 | Q8N3X1, -2 | S | 116;118 | 2.781 | 0.006 |
| PDLIM2 | Q96JY6, -2, -3, -4, -5 | S | 134;384 | -4.135 | 0.006 |
| DENND4C | Q5VZ89, -2, -5, -6 | S | 208;732 | -10.124 | 0.008 |
| GPS1 | Q13098, -5, -7 | S | 470;474;510 | -8.956 | 0.010 |
| PHKB | Q93100, -2, -3, -4 | S | 693;700 | 3.497 | 0.011 |
| CD200R1 | Q8TD46, -4 | S | 279;302 | 7.916 | 0.012 |
| REEP4 | Q9H6H4 | S | 150 | -3.091 | 0.013 |
| PXN | P49023, -2, -3 | S | 126 | 3.103 | 0.014 |
| EEF1D | P29692, -2, -3, -4 | S | 133;499;114;109 | 2.269 | 0.015 |
| PPP1R14A | Q96A00, -2 | S | 101;128 | 2.594 | 0.015 |
| EIF3B | P55884, -2 | S | 117 | 2.275 | 0.015 |
| KIAA0930 | Q6ICG6, -2, -3 | S | 270;304;309 | 6.823 | 0.015 |
| HTT | P42858 | S | 1870 | -6.464 | 0.017 |
| TMPO | P42167, -2, -3 | S | 156 | -2.529 | 0.017 |
| ARRB1 | P49407, -2 | S | 404;412 | 4.343 | 0.018 |
| CEBPB | P17676, -2, -3 | S | 33;208;231 | 5.203 | 0.019 |
| SRRM2 | Q9UQ35, -2 | S | 1382 | -5.971 | 0.019 |
| TOM1 | O60784, -2, -3, -4 | S | 310;322;355 | 2.751 | 0.020 |
| EEF2 | P13639 | T | 54 | -5.759 | 0.021 |
| AKAP2 | Q9Y2D5, -4, -5, -6, -7 | S | 152;241;383 | 5.661 | 0.022 |
| IL16 | Q14005, -2, -3, -4 | S | 94;795 | -2.447 | 0.023 |
| SH3KBP1 | Q96B97, -2, -3 | S | 550;587;349 | 3.580 | 0.023 |
| RCOR1 | Q9UKL0 | S | 257 | -2.444 | 0.023 |
| TMX1 | Q9H3N1 | S | 247 | -2.869 | 0.024 |
| ASAP1 | Q9ULH1, -2 | S | 839;842 | 5.288 | 0.025 |
| FES | P07332, -2, -3, -4 | T | 363;421 | 2.333 | 0.025 |
| NDRG2 | Q9UN36, -2, -3, -4, -5 | S | 307;320;336;339;346;350 | -5.156 | 0.026 |
| HNRNPU | Q00839, -2 | S | 3 | 2.479 | 0.028 |
| MPHOSPH8 | Q99549, -2 | S | 51 | 2.407 | 0.030 |
| FAM122A | Q96E09 | S | 62 | 3.033 | 0.030 |
| DNAJB6 | O75190, -3, -4 | S | 277;228 | 1.906 | 0.030 |
| PGAM1 | P18669 | S | 14 | 2.019 | 0.031 |
| DOCK2 | Q92608 | S | 1731 | -2.210 | 0.031 |
| ZNF609 | O15014 | S | 358 | 2.205 | 0.032 |
| AKAP12 | Q02952, -2, -3 | S | 539;546;644 | -4.607 | 0.032 |
| CEP135 | Q66GS9 | S | 439 | -2.971 | 0.034 |
| FAM21C | Q9Y4E1, -2, -3, -4, -5, -6 | S | 1075;997;1013;1044;1052;1054;979;42 | 2.356 | 0.035 |
| RGS14 | O43566, -4, -5, -6 | S | 288;135;68 | 4.434 | 0.035 |
| MAP1A | P78559, -2 | S | 612 | -4.406 | 0.035 |
| SLTM | Q9NWH9 | S | 289 | 2.530 | 0.037 |
| TBC1D5 | Q92609, -2 | S | 538;560 | 2.518 | 0.037 |
| PPP6R3 | Q5H9R7, -4, -5, -6 | S | 537;588;611;617 | -2.525 | 0.037 |
| ATP6V0A2 | Q9Y487 | S | 695 | 2.423 | 0.039 |
| SH3BP1 | Q9Y3L3 | S | 585 | 1.853 | 0.039 |
| DDB2 | Q92466, -2, -3, -4 | S | 24 | 3.042 | 0.039 |
| MAP1A | P78559;P78559-2 | S | 2018 | -2.392 | 0.040 |
| RAB11FIP1 | Q6WKZ4, -2, -3 | S | 51;199 | -2.347 | 0.041 |
| RGS3 | P49796, -1, -4, -6 | S | 264;662;839;943 | 4.111 | 0.041 |
| EEF1D | P29692, -2, -3, -4 | S | 162;528;143;138 | -4.092 | 0.041 |
| ANKS1A | Q92625 | S | 661 | 4.091 | 0.041 |
| NFATC2IP | Q8NCF5 | S | 204 | 1.967 | 0.042 |
| STUB1 | Q9UNE7 | S | 19 | 4.020 | 0.043 |
| RBM39 | Q14498, -2 | S | 97 | 4.016 | 0.043 |
| MYO1F | O00160 | S | 1001 | 2.279 | 0.043 |
| SERBP1 | Q8NC51, -2, -3, -4 | S | 203 | 3.966 | 0.044 |
| PAK2 | Q13177 | S | 141 | -2.584 | 0.045 |
| MAP7D1 | Q3KQU3, -4 | S | 271 | -3.915 | 0.045 |
| SEC16A | O15027, -2, -3, -4, -5 | S | 1786 | 3.090 | 0.046 |
| PRRC2C | Q9Y520, -2, -3, -4, -5, -6 | S | 1546;1301;1544 | -1.978 | 0.046 |
| ADD1 | P35611, -2, -3 | S | 726;757;713;661;693 | -2.547 | 0.046 |
| SLC27A3 | Q5K4L6, -2, -3 | S | 173 | 2.693 | 0.047 |
| PPIG | Q13427 | S | 397 | 1.863 | 0.048 |
| HNRNPK | P61978, -2, -3 | S | 284;260 | 2.677 | 0.049 |
| TALDO1 | P37837 | S | 237 | -2.048 | 0.050 |

**Sl.Table 5 | Significantly altered phosphoproteins are involved in multiple biological processes.** GO terms enriched in the altered phosphoproteins upon α2-3sia binding in presence of LPS are organized in groups, as seen in [Figure 3A](#Fig3).

| GO Groups | GO ID | GO Term | Group P value | n | Associated Proteins |
| --- | --- | --- | --- | --- | --- |
| SMAD protein signaling | GO:0060395 | SMAD protein signal transduction | 0.00422 | 3 | JUN, RBM14, VIM |
| Podosome assembly | GO:0071800 | podosome assembly | 0.00024 | 3 | ARHGEF2, BIN2, LCP1 |
| Organelle assembly | GO:1902116 | negative regulation of organelle assembly | 0.00059 | 3 | ARHGEF2, MAP4, RBM14 |
| Growth factor response | GO:0070849 | response to epidermal growth factor | 0.00200 | 3 | DNAJC5, NCL, ZFP36 |
|  | GO:0071364 | cellular response to epidermal growth factor stimulus | 0.00200 | 3 | DNAJC5, NCL, ZFP36 |
| mRNA regulation | GO:0050684 | regulation of mRNA processing | 0.00066 | 5 | ACIN1, HNRNPA2B1, IWS1, RBMX, THRAP3 |
|  | GO:1903312 | negative regulation of mRNA metabolic process | 0.00066 | 5 | ACIN1, HNRNPA2B1, RBMX, THRAP3, ZFP36 |
|  | GO:1903313 | positive regulation of mRNA metabolic process | 0.00066 | 3 | RBMX, THRAP3, ZFP36 |
|  | GO:0033119 | negative regulation of RNA splicing | 0.00066 | 3 | ACIN1, HNRNPA2B1, RBMX |
|  | GO:0050686 | negative regulation of mRNA processing | 0.00066 | 3 | ACIN1, HNRNPA2B1, RBMX |
|  | GO:0048024 | regulation of mRNA splicing, via spliceosome | 0.00066 | 4 | ACIN1, HNRNPA2B1, RBMX, THRAP3 |
|  | GO:0048025 | negative regulation of mRNA splicing, via spliceosome | 0.00066 | 3 | ACIN1, HNRNPA2B1, RBMX |
| Growth hormone response | GO:0001938 | positive regulation of endothelial cell proliferation | 4.38E-05 | 4 | JUN, MIA3, STAT3, STAT5A |
|  | GO:0060416 | response to growth hormone | 4.38E-05 | 3 | PXN, STAT3, STAT5A |
|  | GO:0045646 | regulation of erythrocyte differentiation | 4.38E-05 | 3 | MIA3, STAT3, ZFP36 |
|  | GO:0045639 | positive regulation of myeloid cell differentiation | 4.38E-05 | 4 | ACIN1, JUN, MIA3, STAT3 |
|  | GO:0071378 | cellular response to growth hormone stimulus | 4.38E-05 | 3 | PXN, STAT3, STAT5A |
|  | GO:0060396 | growth hormone receptor signaling pathway | 4.38E-05 | 3 | PXN, STAT3, STAT5A |
|  | GO:0009299 | mRNA transcription | 4.38E-05 | 3 | EEF1D, MIA3, STAT3 |
|  | GO:0006110 | regulation of glycolytic process | 4.38E-05 | 3 | EIF6, MIA3, STAT3 |
| Regulation of proliferation | GO:0070671 | response to interleukin-12 | 1.02E-05 | 3 | HNRNPA2B1, LCP1, STAT3 |
|  | GO:0035722 | interleukin-12-mediated signaling pathway | 1.02E-05 | 3 | HNRNPA2B1, LCP1, STAT3 |
|  | GO:0071349 | cellular response to interleukin-12 | 1.02E-05 | 3 | HNRNPA2B1, LCP1, STAT3 |
|  | GO:0034101 | erythrocyte homeostasis | 1.02E-05 | 5 | ACIN1, MIA3, RPS6, STAT3, ZFP36 |
|  | GO:0001938 | positive regulation of endothelial cell proliferation | 1.02E-05 | 4 | JUN, MIA3, STAT3, STAT5A |
|  | GO:0016441 | posttranscriptional gene silencing | 1.02E-05 | 5 | EIF6, HNRNPA2B1, NCOR2, STAT3, ZFP36 |
|  | GO:0030218 | erythrocyte differentiation | 1.02E-05 | 5 | ACIN1, MIA3, RPS6, STAT3, ZFP36 |
|  | GO:0045646 | regulation of erythrocyte differentiation | 1.02E-05 | 3 | MIA3, STAT3, ZFP36 |
|  | GO:0035194 | posttranscriptional gene silencing by RNA | 1.02E-05 | 5 | EIF6, HNRNPA2B1, NCOR2, STAT3, ZFP36 |
|  | GO:0045639 | positive regulation of myeloid cell differentiation | 1.02E-05 | 4 | ACIN1, JUN, MIA3, STAT3 |
|  | GO:0035195 | gene silencing by miRNA | 1.02E-05 | 5 | EIF6, HNRNPA2B1, NCOR2, STAT3, ZFP36 |
|  | GO:0045974 | regulation of translation, ncRNA-mediated | 1.02E-05 | 3 | EIF6, STAT3, ZFP36 |
|  | GO:0009299 | mRNA transcription | 1.02E-05 | 3 | EEF1D, MIA3, STAT3 |
|  | GO:0040033 | negative regulation of translation, ncRNA-mediated | 1.02E-05 | 3 | EIF6, STAT3, ZFP36 |
|  | GO:0035278 | miRNA mediated inhibition of translation | 1.02E-05 | 3 | EIF6, STAT3, ZFP36 |
|  | GO:0006110 | regulation of glycolytic process | 1.02E-05 | 3 | EIF6, MIA3, STAT3 |

**Sl.Table 6 | Significantly altered phosphoproteins are involved in five biological processes.** GO terms enriched in the altered phosphoproteins upon α2-6sia binding in presence of LPS are organized in groups, as seen in [Sl. Figure 3](#SlFig3).

| GO ID | GO Term | Group P value | n | Associated Proteins |
| --- | --- | --- | --- | --- |
| GO:0043154 | negative regulation of cysteine-type endopeptidase activity involved in apoptotic process | 0.0005 | 4 | ARRB1, DNAJB6, PAK2, RAF1 |
| GO:0043407 | negative regulation of MAP kinase activity | 0.0026 | 3 | GPS1, RGS14, RGS3 |
| GO:0048260 | positive regulation of receptor-mediated endocytosis | 0.0011 | 3 | ARRB1, HNRNPK, TBC1D5 |
| GO:0070373 | negative regulation of ERK1 and ERK2 cascade | 0.0018 | 3 | ARRB1, NDRG2, RGS14 |
| GO:1902117 | positive regulation of organelle assembly | 0.0021 | 3 | ASAP1, CEP135, HTT |
